# Decoding neuro-tumor interactions in pancreatic cancer: mechanisms, immunosuppressive networks and therapeutic opportunities

**DOI:** 10.1186/s43556-026-00586-2

**Published:** 2026-09-17

**Authors:** Wei Cheng, Haotian Cao, Hao Hu, Rui Qian, Bingjie Xu, Jing Zhang

**Affiliations:** 1 Department of Pathology, The Second Affiliated Hospital of Naval Medical University, Shanghai, 200003 China; 2 Department of Pathology, The First Affiliated Hospital of Naval Medical University, Shanghai, 200003 China

**Keywords:** Pancreatic ductal adenocarcinoma, Perineural invasion, Neuro-immune axis, Therapeutic strategy

## Abstract

Pancreatic cancer is one of the most lethal digestive system malignancies worldwide, characterized by insidious onset, limited early detection approaches and extremely poor long-term prognosis. It comprises multiple histological subtypes, among which pancreatic ductal adenocarcinoma (PDAC) represents the most prevalent exocrine neoplasm and accounts for the vast majority of all pancreatic cancer cases. Perineural invasion (PNI) stands as one of the defining biological hallmarks of PDAC. Detected in 80%–100% of PDAC patients, PNI serves as an independent adverse prognostic factor that drives local recurrence, distant metastasis, and resistance to both chemotherapy and immunotherapy. Historically regarded as a passive route for tumor dissemination, PNI is now recognized as a complex, multicellular process involving bidirectional crosstalk between cancer cells, nerve fibers, stromal cells, and immune components within the tumor microenvironment. This review dissects the molecular mechanisms underlying neuro-tumor interactions and proposes a unified four-stage mechanistic model of PNI, encompassing mutual chemotaxis between tumors and nerves, adhesion and invasion at the tumor-nerve interface, extracellular matrix remodeling, and neural plasticity alterations. Notably, we define the perineural invasion microenvironment as a neuro-immune privileged sanctuary, and delineate its multicellular composition and pathological consequences based on current research advances. Furthermore, we summarize potential therapeutic strategies targeting the neuro-immune-tumor axis and ongoing clinical trials, discuss key translational challenges, and highlight the transformative applications of multi-omics technologies and artificial intelligence in PNI diagnosis, mechanistic discovery, and therapeutic optimization. This work provides a comprehensive theoretical framework for understanding PNI mechanisms and guides future translational research and precision therapy development for pancreatic cancer.

## Introduction

Pancreatic ductal adenocarcinoma (PDAC) is the most prevalent subtype of pancreatic cancer, hallmarked by aggressive biological behavior, rapid disease progression, late-stage diagnosis, and an extremely poor prognosis [1, 2]. Population-based statistics indicate that the 5-year overall survival rate for all pancreatic cancer patients remains merely 13%, whereas the median overall survival is only approximately 4 to 6 months specifically in patients with untreated metastatic PDAC, rendering it one of the most lethal malignancies of the digestive system [3, 4]. Perineural invasion (PNI), a prominent pathological hallmark of PDAC, is a key driver of the high recurrence and metastasis rates observed in this disease [5, 6]. PNI serves as an independent prognostic marker and plays a central role in the development of adverse clinical outcomes in PDAC [7]. Notably, PDAC exhibits the highest incidence of PNI among all solid tumors; histopathological analysis of whole-mount serial sections from PDAC specimens demonstrates that the detection rate of perineural invasion can reach 80% to 100% [8, 9].

Historically, nerves were merely regarded as passive mechanical conduits for cancer cell dissemination [10]. However, accumulating evidence has reshaped this paradigm: PNI is not merely an anatomical route for tumor spread, but a specialized, immunosuppressive perineural sanctuary constructed through dynamic, bidirectional crosstalk among tumor cells, nerve fibers, Schwann cells, cancer-associated fibroblasts, and diverse immune populations including tumor-associated macrophages, myeloid-derived suppressor cells, regulatory T cells, and exhausted CD8^+^ T cells [11–13]. This multicellular perineural niche drives not only local tumor invasion and distant metastasis, but also cancer-associated pain, systemic immune evasion, and resistance to chemotherapy and immunotherapy, collectively underlying the dismal prognosis of PDAC [7]. Key seminal discoveries underpinning this framework include: (1) Schwann cells undergo reprogramming in response to tumor invasion, forming migratory tracks that guide cancer cell dissemination and secreting soluble factors to remodel the local microenvironment; (2) tumor-associated nerve fibers release neurotransmitters and chemokines that actively recruit and polarize immunosuppressive cells, establishing an immune-excluded microenvironment around invaded nerves; (3) cancer-associated fibroblasts and pancreatic stellate cells collaborate with tumor cells to remodel the extracellular matrix, creating a physical and biochemical permissive niche that sustains PNI progression. Furthermore, a landmark 2025 Nature study confirmed that cancer-induced nerve injury directly drives resistance to anti-PD-1 immunotherapy, establishing a causal link between perineural niche formation and therapeutic failure in pancreatic cancer [14].

While existing reviews have systematically catalogued individual signaling axes and sequential pathological steps of PNI, a unified conceptual framework that integrates neural plasticity, stromal remodeling, and immunosuppression into a coherent perineural sanctuary model remains to be fully articulated. This paradigm shift in understanding has not only deepened mechanistic insights into PDAC progression, but also positioned targeted disruption of the neuro-immune-tumor axis as a promising therapeutic avenue to overcome treatment resistance.

**Fig. 1:**
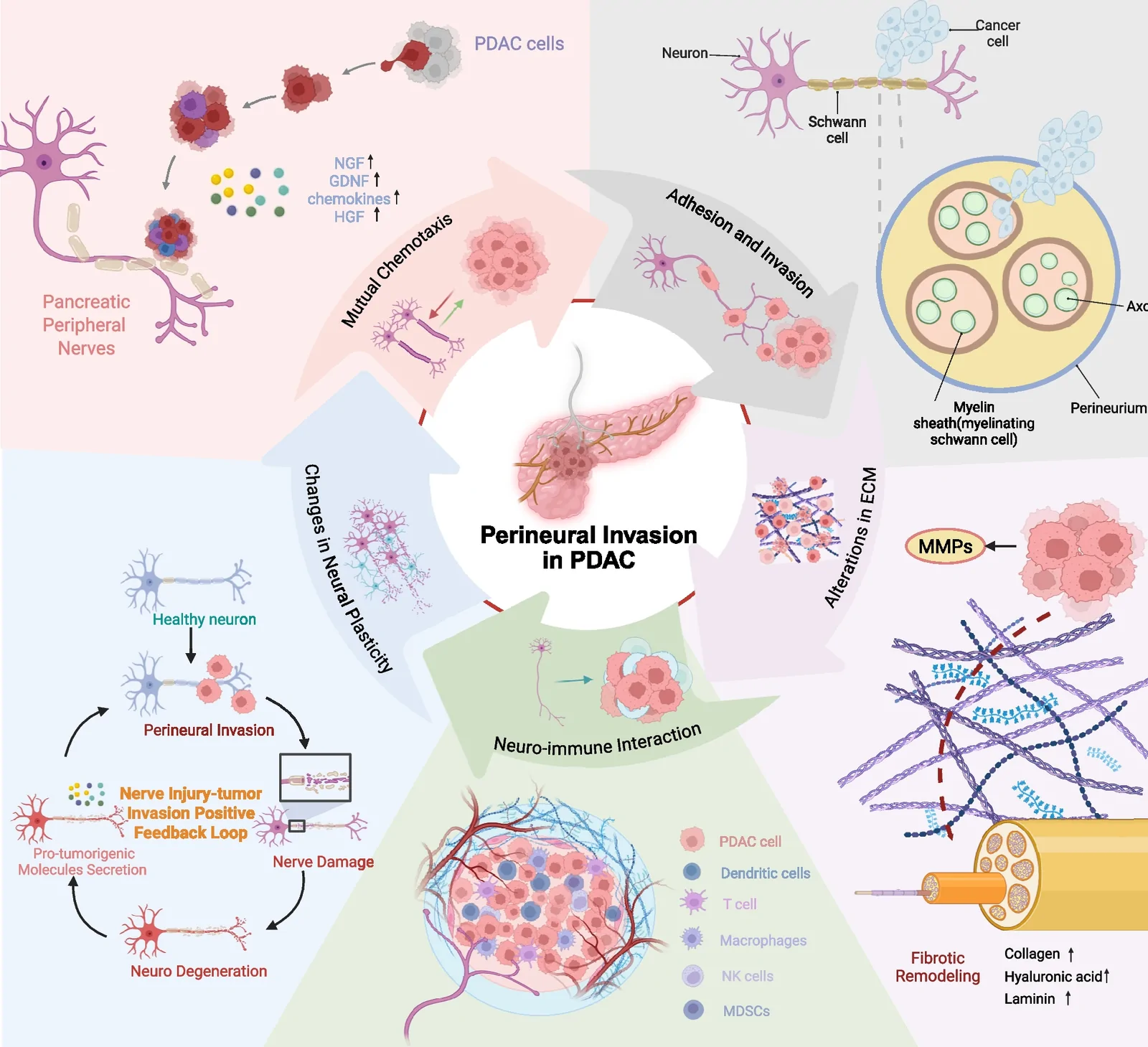
Processes of PNI in PDAC. This figure elucidates four key processes of PNI in PDAC: mutual chemotaxis between tumors and nerves, adhesion and invasion at the tumor-nerve interface, alterations in the extracellular matrix, and changes in neural plasticity. Concurrently, the immune microenvironment of the tumor is also undergoing modifications. NGF: Nerve Growth Factor. GDNF: Glial Cell Line-Derived Neurotrophic Factor. MMP: Matrix Metalloproteinase. NK: Natural Killer. MDSC: Myeloid-Derived Suppressor Cell

Building on the latest advances in the field, this review has three core objectives, with a central focus on elaborating the immunosuppressive perineural sanctuary as its core conceptual contribution. First, we dissect the multilayered mechanisms of PDAC-associated PNI and organize the sequential pathological processes into a four-stage model (Fig. 1): (1) reciprocal chemotaxis between tumor and nerve compartments that initiates PNI; (2) adhesion and basement membrane invasion at the tumor-nerve interface; (3) perineural extracellular matrix remodeling; and (4) adaptive neural plasticity. In parallel, we systematically delineate the cellular and molecular architecture of the immunosuppressive perineural niche and its functional consequences. Second, we review the current landscape of therapeutic strategies targeting the neuro-immune-tumor axis, explicitly distinguishing interventions directly acting on PNI pathways from those with indirect neuroimmune modulatory effects, and discuss clinically actionable translational opportunities. Finally, we synthesize cutting-edge progress in multi-omics and artificial intelligence applied to PNI research, outline prevailing challenges in the field, and propose a prioritized roadmap for future investigation.

## Neuroanatomical basis in normal pancreas and perineural invasion

The pancreas is one of the most densely innervated organs in the human body, with a complex neural network that regulates exocrine secretion, endocrine function, and pain perception [15, 16]. This intricate innervation system not only maintains physiological homeostasis but also provides the anatomical foundation for the exceptionally high incidence of PNI in pancreatic cancer.

While pancreatic ductal adenocarcinoma represents the best-characterized pancreatic malignancy with respect to PNI—exhibiting a 80%–100% incidence and well-elucidated molecular, cellular, and immunological underpinnings—PNI profiles across other pancreatic neoplasms remain markedly understudied and exhibit pronounced histotype-specific heterogeneity [17]. Pancreatic neuroendocrine neoplasms (PanNENs) display an overall PNI prevalence of 12%–24.4%, which increases with tumor grade and independently predicts inferior disease-free and overall survival following surgical resection [18]. For intraductal papillary mucinous neoplasms (IPMN), PNI is exceedingly rare in non-invasive lesions even with high-grade dysplasia, while IPMN-associated invasive carcinomas harbor lower PNI frequency than sporadic PDAC; notably, intrapancreatic nerve density and accumulation of immature neural progenitors peak at the high-grade dysplasia stage, preceding the onset of stromal invasion [19]. Solid pseudopapillary neoplasms, as indolent tumors of low malignant potential, only rarely exhibit focal PNI in subsets with locally aggressive histological features [20]. Collectively, mechanistic paradigms and therapeutic evidence for PNI have been almost exclusively established in PDAC.

The pancreas receives dual innervation from the extrinsic autonomic nervous system and the intrinsic enteric nervous system, forming a highly integrated regulatory network [21]. The parasympathetic innervation of the pancreas primarily originates from preganglionic neurons located in the dorsal motor nucleus of the vagus nerve in the medulla oblongata [22]. These preganglionic fibers travel along the vagus nerve and synapse with postganglionic neurons within the intrapancreatic ganglia. Postganglionic parasympathetic fibers release acetylcholine (ACh) and vasoactive intestinal peptide, stimulating pancreatic exocrine secretion and insulin release from beta cells [23]. Neural tracing studies have demonstrated that distinct branches of the vagus nerve selectively innervate the exocrine and endocrine compartments of the pancreas, exhibiting remarkable functional specificity [24]. In contrast, the sympathetic innervation arises from preganglionic neurons in the intermediolateral column of the thoracolumbar spinal cord (T5-L2). These fibers project via the splanchnic nerves to the celiac and superior mesenteric ganglia, where they synapse with postganglionic neurons. Postganglionic sympathetic fibers release norepinephrine and neuropeptide Y, inhibiting pancreatic exocrine secretion and regulating vascular tone [25, 26]. Importantly, these neurotransmitters are not only involved in physiological regulation but also serve as key signaling molecules mediating tumor-neural crosstalk, providing the molecular basis for PNI initiation [27]. In addition to extrinsic innervation, the pancreas contains an extensive intrinsic neural network composed of approximately 500,000 neurons organized into intrapancreatic ganglia. This intrinsic system includes sensory neurons, interneurons, and motor neurons that form local reflex arcs capable of regulating pancreatic function independently of central nervous system input [28, 29]. Emerging evidence indicates that the intrinsic pancreatic nervous system undergoes early remodeling during pancreatic carcinogenesis, serving as the initial target for tumor cell invasion even before the development of invasive carcinoma [30].

The extrapancreatic nerve plexuses represent the primary pathways for pancreatic cancer cells to spread beyond the pancreatic parenchyma [31]. These plexuses are anatomically divided into six major components, each with distinct clinical implications for tumor dissemination and surgical resection [32]. Pancreatic head plexus I originates from the right side of the celiac ganglion and courses along the superior pancreaticoduodenal artery to the posterior surface of the pancreatic head. Approximately 70% of pancreatic head cancers invade this plexus, making it the most commonly affected extrapancreatic neural structure. Pancreatic head plexus II extends from the bilateral celiac ganglia to the left margin of the uncinate process, running in close proximity to the superior mesenteric artery. Complete resection of this plexus is technically challenging due to its intimate association with major blood vessels, and residual tumor in this plexus is the leading cause of early local recurrence after pancreaticoduodenectomy. The celiac plexus, located around the origin of the celiac trunk, is the largest autonomic plexus in the abdominal cavity. Celiac plexus invasion is associated with severe abdominal pain and a particularly poor prognosis [33]. The superior mesenteric artery plexus encircles the superior mesenteric artery and extends along its branches, and invasion of this plexus is considered a contraindication to curative resection in most clinical guidelines [34]. The hepatic plexus runs along the common hepatic artery and portal vein, and its invasion is associated with an increased risk of liver metastasis. The splenic plexus follows the splenic artery to the splenic hilum, serving as the primary route of neural dissemination for cancers arising in the pancreatic body and tail, and its invasion is strongly correlated with retroperitoneal recurrence and metastasis [30, 35, 36].

Perineural invasion is the defining pathological hallmark of PDAC and a critical determinant of its aggressive clinical behavior. Perineural invasion is the process by which cancer cells invade and spread along nerves, defined as tumor cells surrounding at least 33% of the circumference of a nerve or invading any of the three layers of the nerve sheath (endoneurium, perineurium, or epineurium) [37]. The nerve sheath consists of three concentric layers with distinct barrier functions: the epineurium is the outermost layer composed of loose connective tissue, which is the first barrier breached by invading tumor cells; the perineurium is a multilayered structure of specialized epithelial cells that forms a tight mechanical and biological barrier, and its disruption is a key step in the progression of PNI; the endoneurium is the innermost layer surrounding individual axons, and once tumor cells penetrate this layer, they can spread rapidly along the axonal space over long distances [17, 38, 39]. This invasion precipitates a spectrum of neurological manifestations including intractable pain, sensory deficits (numbness), and motor weakness, all of which significantly compromise treatment efficacy and severely impair patients' quality of life. PNI is prevalent in malignancies arising from densely innervated anatomical regions—including the head and neck [40], lung [41], stomach [42], colon [43], and rectum [44] —and is now established as a defining pathological hallmark with profound prognostic implications across multiple solid tumor types. PNI in pancreatic cancer is associated with characteristic histopathological changes, including neural hypertrophy, increased neural density, and neuritis [45]. Growth-associated protein 43 (GAP-43) and glial fibrillary acidic protein (GFAP) are well-established immunohistochemical markers for assessing neural remodeling, with their expression levels correlating directly with the severity of PNI and patient prognosis [45, 46].

During PNI, PDAC cells undergo a phenotypic transition in their migratory behavior, adopting an amoeboid migration mode characterized by rounded cell morphology, prominent membrane blebbing, and enhanced motility at the leading edge of nerve invasion [47]. Importantly, PNI is not solely driven by intrinsic properties of cancer cells but is actively supported by various non-malignant cellular components within TME. Transcriptomic profiling of PDAC nerve bundles containing tumor-associated macrophages (TAMs) isolated from KPC mouse models has identified upregulated expression of C–C motif chemokine ligand 7 (CCL7), C-X-C motif chemokine ligand 12 (CXCL12), and CXCL13 as key mediators of TAM chemotaxis to damaged nerves during PNI progression [48]. Recent studies utilizing murine sciatic nerve invasion models and KPC mice have further demonstrated that Cancer-Associated Fibroblasts (CAFs) promote PDAC PNI by transferring the long noncoding RNA (lncRNA) *PIAT* via extracellular vesicles [49]. Mechanistically, *PIAT* stabilizes YBX1 protein, thereby modulating mRNA stability and stimulating the expression of PNI-related genes through m5C epigenetic modification. The detailed molecular mechanisms underlying PNI will be elaborated in the next chapter.

## Mechanisms of perineural invasion in PDAC

Based on previous studies, we summarize the known cellular and molecular events occurring during PNI in PDAC and propose a unified four-stage mechanistic model that fully recapitulates the entire process of neurotropic invasion from initiation to progression: (1) mutual chemotaxis between tumors and nerves, (2) adhesion and invasion at the tumor-nerve interface, (3) alterations in the extracellular matrix, and (4) changes in neural plasticity.

Notably, these four stages are not temporally segregated but operate synergistically in a dynamic and interdependent manner, forming a vicious cycle that drives the progression of perineural invasion, tumor metastasis, and the development of therapeutic resistance. Shared signaling nodes such as nerve growth factor (NGF), transforming growth factor-β (TGF-β), and matrix metalloproteinases (MMPs) play critical regulatory roles across multiple stages, representing high-priority therapeutic targets with significant translational potential. Meanwhile, the immune microenvironment of PDAC also engages in reciprocal interactions with neurons: neurons can respond to signaling molecules from the immune system, while immune cells react to neurotransmitters and neuromodulators. This crosstalk is also a central focus of the present review.

### Mutual chemotaxis of tumor-nerve axis promotes perineural invasion

**Fig. 2:**
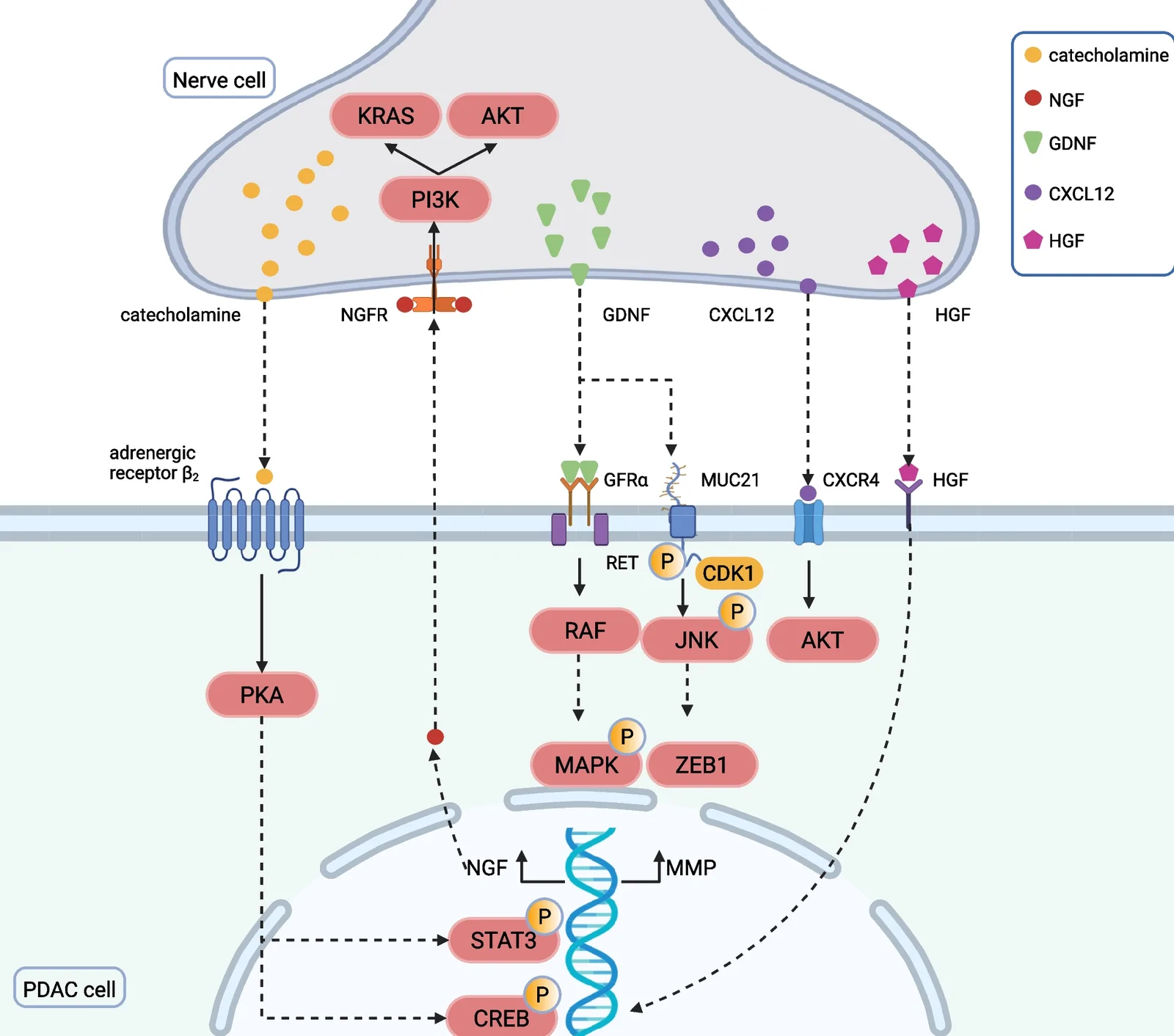
Signaling molecules in neuro—tumor mutual chemotaxis. Between tumors and nerves, mutual chemotaxis occurs via signaling molecules including catecholamine, NGF, GDNF, CXCL12, and HGF. Catecholamine binds to the adrenergic receptor β₂ on PDAC cells, activating PKA. NGF binds NGFR, triggering the PI3K—AKT pathway. GDNF binds GFRα/RET, activating RAF and MAPK. CXCL12 interacts with CXCR4, influencing AKT. HGF binds its receptor, affecting AKT and other pathways. Intracellular signaling molecules like KRAS, STAT3, and CREB regulate gene expression (e.g., MMP). This illustrates the complex signaling crosstalk driving PDAC—nerve interactions, with pathways such as PI3K—AKT, MAPK, and JNK playing key roles in this communication

The mutual chemotaxis between tumors and nerves is driven by various factors, including NGF [50], GDNF [51], chemokines, hepatocyte growth factor (HGF) [52], and other bioactive molecules. The combined effect of these factors is to enhance the bidirectional chemotactic signaling between tumors and nerves so as to promote perineural invasion (Fig. 2).

NGF has been demonstrated to be generated in both PDAC cells and nerves, with both expressing their respective receptors [53]. The nerve growth factor family encompasses NGF, brain-derived neurotrophic factor (BDNF), neurotrophic factor-3 (NT-3), and neurotrophic factor-4 (NT-4) [54]. NGF is capable of binding to the highly specific TrkA receptor as well as the less specific p75NTR receptor [55]. The binding of NGF to the TrkA receptor induces the phosphorylation of TrkA, thereby activating the PI3K-Akt or Ras-MAPK pathways and facilitating cell survival and proliferation [56]. When NGF binds to the p75NTR receptor, p75NTR may have two opposing effects. It can recruit the tumor necrosis factor receptor member TRAF6 and activate NF-κB signaling to promote cell survival, or it can mediate the JNK pathway and activate PTEN to inhibit growth and promote apoptosis [57, 58]. On the one hand, NGF plays a significant role in the attraction of neurons by tumor cells and the maintenance of neurons [59, 60]. On the other hand, the role of NGF in promoting the invasion of tumor cells into nerve cells as well as tumor proliferation and migration has also been confirmed [50, 61]. As early as the pancreatic intraepithelial neoplasia (PanIN) stage, a marked increase in the mRNA expression of pancreatic neurotrophic factors and sensory innervation has been observed, highlighting the significant role of neurotrophic factors in tumorigenesis [62]. The binding of NGF to the p75 neurotrophin receptor (p75NTR) activates the NF-κB signaling pathway, which plays a crucial role in tumorigenesis and tumor invasion. Notably, the use of NF-κB pathway inhibitors has been shown to effectively suppress NGF-mediated perineural invasion in tumors [63, 64]. Studies have demonstrated that blocking NGF signaling through siRNA or small-molecule inhibitors targeting p75 effectively inhibits the migration of tumor cells toward nerves [65]. Furthermore, pancreatic cancer cells have been found to secrete NGF under energy-deprived conditions, which stimulates axonal secretion of serine by neuronal cells, thereby providing metabolic support for the cancer cells [66].

The GDNF family also plays a role in promoting the reciprocal chemotaxis between nerve cells and cancer cells. The GDNF family of ligands includes GDNF, neurturin, artemin, and persephin [54]. Members of the GDNF family bind to the glycosylphosphatidylinositol-anchored GDNF family receptor α1 (GFRα1) [67]. GFRα1 recruits the rearranged receptor tyrosine kinase during the transfection process for dimerization [68]. Recent studies have revealed that neurotrophic factors phosphorylate the intracellular domain S543 of MUC21 through cyclin-dependent kinase 1 (CDK1) in PDAC cells, which promotes the interaction between MUC21 and RAC2 [51]. This interaction leads to membrane anchoring and the activation of RAC2, which in turn activates the c-Jun N-terminal kinase (JNK)/zinc finger E-box binding homeobox 1 (ZEB1)/epithelial-mesenchymal transition (EMT) axis. Ultimately, this enhances the metastasis and PNI of PDAC cells. Therefore, this indicates that MUC21 is a potential prognostic biomarker and therapeutic target for PDAC. Another interesting recent finding is that long non-coding RNAs promote the expression of GDNF by regulating the enrichment of E2F transcription factor 1 (E2F1) and histone acetyltransferase p300 in the promoter region of GDNF, thereby facilitating the perineural invasion of pancreatic cancer [69]. Investigating the GDNF-related histone modifications is also helpful for us to understand the mechanisms underlying the phenomenon of perineural invasion in pancreatic cancer.

In addition to neurotrophic factors, chemokine ligands and their associated receptors can also promote PNI in pancreatic cancer [70]. The CXCL12-CXCR4 axis has been demonstrated to exert pro-tumorigenic effects in various cancers, including gastric [71] and colorectal cancers [72], by facilitating cancer cell proliferation, metastasis, and angiogenesis. In pancreatic cancer, the primary source of CXCL12 is CAFs [73]. Pancreatic cancer cells promote CXCL12 production by CAFs through the secretion of factors such as TNF-α and TGF-β [74]. CXCL12 binds to two cell surface receptors, CXCR4 and ACKR3, activating downstream signaling pathways including phospholipase C, MAPK, PI3K-Akt-mTOR, and JAK/STAT, thereby driving tumor growth and invasion [75]. Furthermore, CX3CR1 and its unique ligand CX3CL1 have been shown to promote tumor cell migration toward nerve cells in pancreatic cancer [76].

The intricate interplay between tumors and nerves, mediated by neurotrophic factors, chemokines, and associated signaling pathways, underscores the complexity of PNI in PDAC. Key molecules such as NGF and GDNF not only facilitate bidirectional chemotaxis but also activate downstream pathways that drive tumor survival, migration, and neural remodeling [55, 64]. Notably, the dual roles of NGF via TrkA and p75NTR receptors highlight context-dependent therapeutic opportunities, while GDNF-mediated MUC21-RAC2 axis activation and lncRNA-regulated GDNF expression unveil novel prognostic and therapeutic targets [51, 57]. Additionally, chemokine networks like CXCL12-CXCR4 and CX3CL1-CX3CR1 further amplify tumor-nerve crosstalk by engaging stromal components such as CAFs [75, 76]. These findings collectively emphasize the potential of targeting neurotrophic and chemokine signaling to disrupt PNI. Future studies should explore the translational feasibility of these targets and investigate how epigenetic modifications or metabolic symbiosis shape the tumor-neural niche, thereby refining strategies to combat neural-driven metastasis in pancreatic malignancies.

### Adhesion and invasion at the tumor-nerve interface

Peripheral nerves are composed of nerve fibers, each consisting of axons enveloped by Schwann cells, which aggregate to form nerve bundles [7]. These structures are protected by three concentric layers of connective tissue: the endoneurium, perineurium, and epineurium [77]. These connective tissues are enriched with ECM components such as laminin, fibronectin, and type IV collagen [78]. Under the influence of various neurotrophic factors and chemokines, tumor cells migrate toward nerve cells and adhere to nerve fibers. Subsequently, through coordinated interactions with surrounding stromal cells (e.g., cancer-associated fibroblasts, immune cells), tumor cells initiate invasive processes into the nerves, leading to PNI [79].

**Fig. 3:**
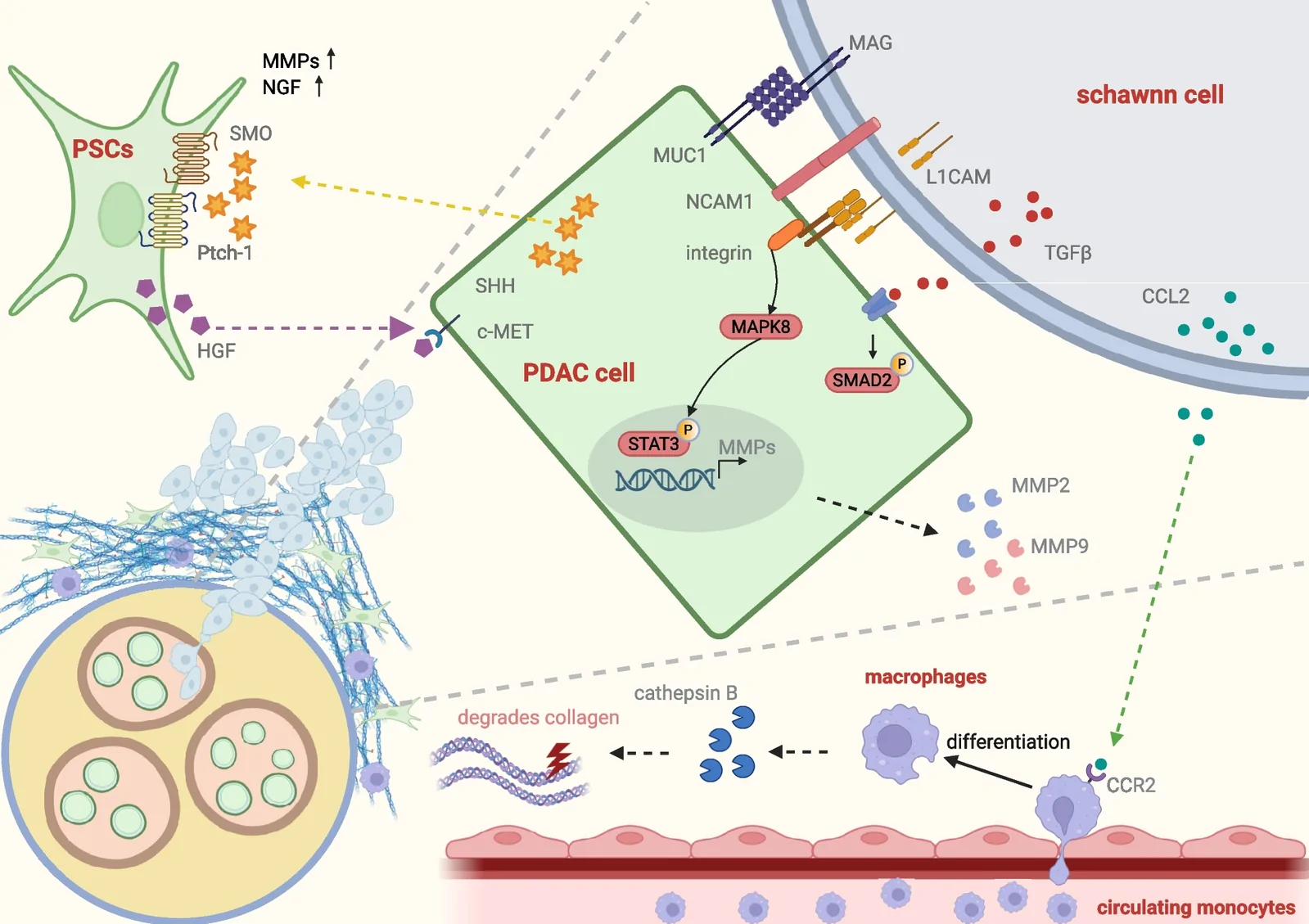
Schematic diagram of PDAC adhesion and invasion of nerves. When tumor cells come into contact with Schwann cells, NCAM-1, MAG, and L1CAM expressed by Schwann cells promote adhesion with tumor cells. Meanwhile, TGF-β secreted by Schwann cells activates the SMAD signaling pathway to enhance tumor nerve invasion. Secreted CCL2 recruits CCR2 monocytes to secrete cathepsin B. Cathepsin B and MMP secreted by the tumor degrade the extracellular matrix to aid tumor movement. SHH and HGF secreted by PSCs jointly up-regulate the expression of NGF and MMP, jointly promoting the adhesion and invasion of PDAC cells to nerves. NCAM-1: Neural Cell Adhesion Molecule 1. MAG: Myelin-Associated Glycoprotein. L1CAM: L1 Cell Adhesion Molecule. CCR: C–C motif chemokine receptor

When discussing tumor adhesion and invasion into intrapancreatic nerves, it is essential to investigate the molecular interactions occurring at the tumor-nerve interface. Schwann cells, as the glial cells in the peripheral nervous system, ensheathe nerve fibers to form the myelin sheath while supporting neuronal survival and promoting axonal regeneration [80, 81]. However, emerging evidence reveals that Schwann cells express neural cell adhesion molecule-1 (NCAM-1), which orchestrates tumor cell migration toward neural structures and facilitates direct tumor-neural contact [82]. This molecular guidance mechanism significantly potentiates perineural invasion by malignant cells through biochemical crosstalk at the tumor-nerve microenvironment [83]. At the tumor-nerve interface, Schwann cells express NCAM-1, which enhances intercellular adhesion via cadherins, while concurrently producing myelin-associated glycoprotein (MAG) and L1 cell adhesion molecule (L1CAM). MAG binds to transmembrane mucin MUC1 on tumor cells, thereby promoting tumor adhesion [84]. Meanwhile, L1CAM functions as a potent chemoattractant for cancer cells by activating mitogen-activated protein (MAP) kinase signaling pathways [85]. Collectively, these molecular interactions orchestrate tumor cell adhesion to pancreatic nerve cells (Fig. 3).

In addition to promoting tumor cell adhesion, certain proteins and molecules secreted by Schwann cells exacerbate neural injury and potentiate tumor invasiveness. Schwann cells secrete the chemokine C–C motif chemokine ligand 2 (CCL2), which recruits CCR2⁺ monocytes from the systemic circulation to sites of perineural invasion [86, 87]. These CCR2⁺ monocytes subsequently differentiate into macrophages and secrete cathepsin B, a protease that degrades collagen IV within the perineurium [88]. Preclinical studies have demonstrated that Schwann cells secrete substantial amounts of transforming TGF-β, which activates the TGF-β-SMAD signaling pathway in pancreatic cancer cells, thereby enhancing their neural invasive capacity [89, 90]. In addition to facilitating tumor cell adhesion, NCAM-1 secreted by Schwann cells induces the production of matrix metalloproteinases MMP2 and MMP9 via the STAT3 signaling pathway, further exacerbating tumor-mediated nerve invasion [91, 92].

In normal tissues, one of the most critical roles of Schwann cells is mediating the regeneration and repair of peripheral neurons [93, 94]. Following peripheral nerve injury, Schwann cells undergo phenotypic transition into a repair state, activating myelin autophagy, recruiting macrophages to facilitate clearance of damaged myelin, and forming Büngner bands to guide axonal regeneration [95, 96]. However, in the context of pancreatic cancer-mediated nerve invasion, this neuroregenerative function of Schwann cells paradoxically contributes to pathological progression [97]. On one hand, injured nerve fibers release abundant neurotrophic factors that attract additional cancer cells. On the other hand, Schwann cells generate dynamic guidance tracks conducive to tumor metastasis, enabling cancer cells to migrate along these pathways. This establishes a self-perpetuating cycle: tumor cells invade nerves, nerve injury recruits more tumor cells to exacerbate invasion and migration, and Schwann cells further amplify metastatic spread. Targeting this vicious cycle —by disrupting tumor-nerve crosstalk and redirecting Schwann cell activity toward protective mechanisms—represents a promising avenue to mitigate perineural invasion in pancreatic cancer.

PSCs, as key stromal components, also potentiate the neural invasive capacity of pancreatic cancer cells. Tumor-derived Sonic Hedgehog (SHH) activates the Hedgehog signaling pathway in PSCs, leading to upregulation of matrix metalloproteinases (MMP2 and MMP9) and NGF [98]. Additionally, hepatocyte growth factor (HGF) secreted by PSCs binds to the c-Met receptor on tumor cells, further elevating NGF and MMP-9 expression [99, 100]. Studies demonstrate that siRNA-mediated blockade of the HGF/c-Met pathway significantly attenuates the neural invasive capacity of pancreatic cancer cells [101].

### Alterations in the extracellular matrix

**Fig. 4:**
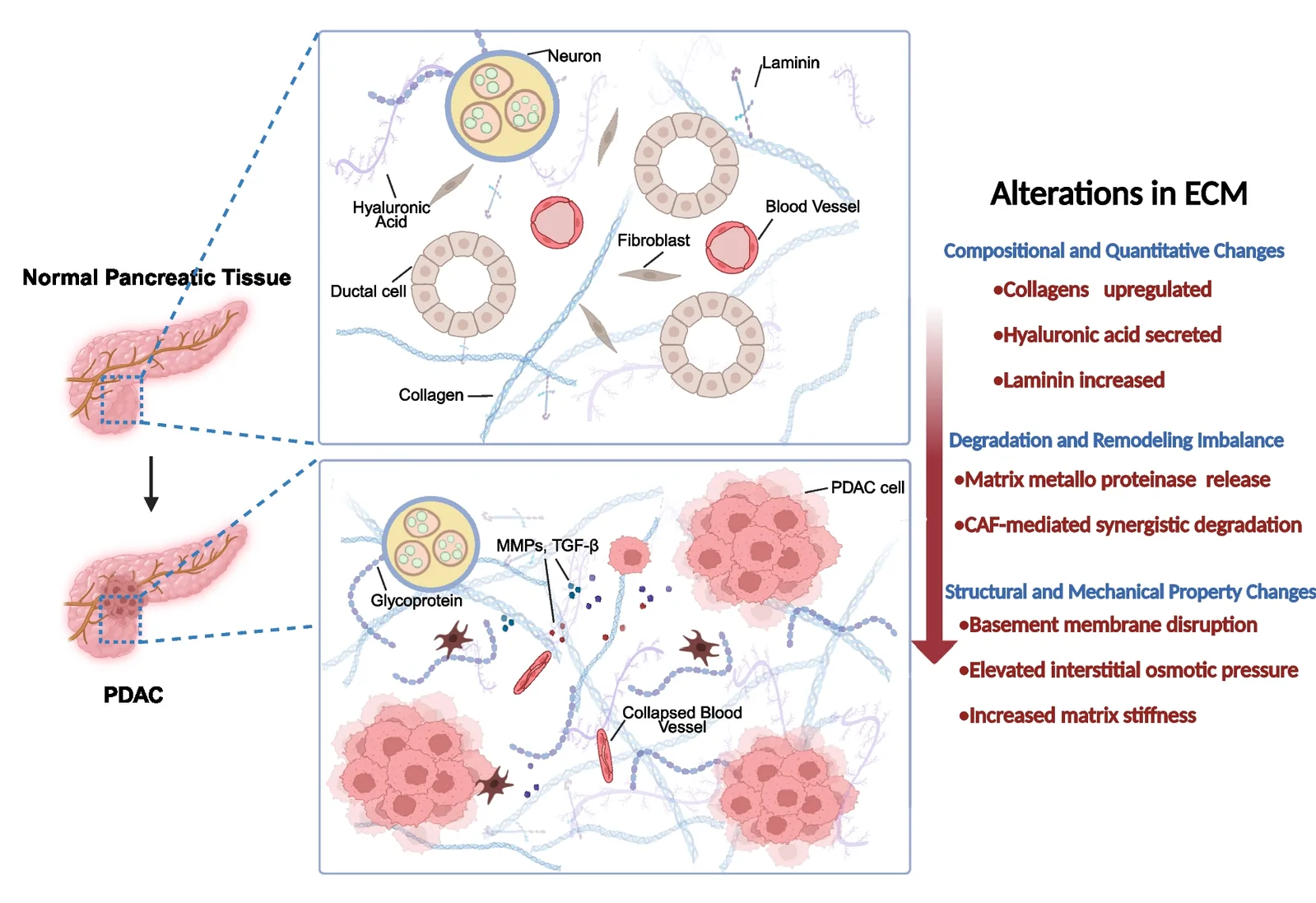
Alteration of the extracellular matrix in PDAC. Compared with the extracellular matrix of normal pancreatic cells, the most remarkable change in PDAC is the matrix pro-fibrotic transformation. Stimulated by TGF-β, CAFs are activated, and collagen types I and III increase nearly threefold, while the amounts of hyaluronic acid, laminin, and glycoprotein also increase significantly. Excessive deposition of collagen and hyaluronic acid leads to increased stiffness of TME and elevated solid stress, causing vascular collapse and ultimately resulting in an acidic and hypoxic TME. PDAC cells release MMPs to degrade the basement membrane, creating low-resistance pathways between cancer cells and neural structures to remove the influence of extracellular matrix deposition

During tumorigenesis, tumor cell chemotaxis toward nerves, and subsequent neural invasion, ECM remodeling occurs concurrently. Notably, studies have demonstrated that ECM alterations emerge as early as the intraepithelial neoplasia stage [102]. The ECM is a dynamic scaffold composed of collagens, proteoglycans, glycoproteins, matricellular proteins, and glycosaminoglycans [103]. It is broadly categorized into two types: the interstitial connective tissue matrix and the basement membrane. Both interstitial and basement membrane matrices contain diverse collagens, including type I and type IV collagens, which enhance tissue tensile strength, promote cell adhesion and chemotaxis, and facilitate migration [102, 104]. Fibronectin, albeit present in smaller quantities, plays a pivotal role in cell adhesion and signal transduction. Similarly, laminin provides critical support for cell adhesion and migration. During pancreatic cancer progression, profound changes in ECM composition and content occur, and this matrix remodeling establishes a favorable microenvironment for tumor growth and metastatic dissemination (Fig. 4).

A hallmark of ECM remodeling during EMT is the pro-fibrotic transformation of the matrix [105]. The original interstitial connective tissue matrix and basement membrane are replaced by a fibrotic ECM dominated by markedly increased collagen, hyaluronic acid (HA), and laminin [106, 107]. CAFs are the primary drivers of this pathological remodeling. CAFs predominantly originate from activated PSCs and tissue-resident fibroblasts [108, 109]. Under stimulation by TGF-β, PSCs and fibroblasts undergo activation [110]. These CAFs overproduce collagens, with type I and III collagens increasing nearly threefold, while tumor cells generate type IV collagen [111, 112]. These collagens bind to integrins on tumor cell surfaces, facilitating proliferation and migration [113]. Furthermore, HA secreted by tumor and stromal cells activates the PI3K-AKT, RhoA, and RAS signaling pathways via the CD44 receptor, promoting tumor cell survival and invasion [114]. The deposition of excessive collagen and HA leads to increased TME stiffness and elevated solid stress, resulting in denser ECM with reduced porosity [107, 115, 116]. HA accumulation also raises interstitial osmotic pressure, causing vascular collapse and ultimately fostering an acidic, hypoxic TME [117, 118]. Collectively, these ECM alterations profoundly reshape the tumor microenvironment, creating a permissive niche for cancer progression.

The deposition of ECM and tissue fibrosis fail to impede cancer cell migration, largely due to the activity of MMPs. During migration through the ECM toward nerves, pancreatic cancer cells release MMPs to degrade the basement membrane, creating low-resistance pathways between cancer cells and neural structures. Multiple cytokines and proteins enhance MMP expression in this process, including GDNF [119], L1-CAM expressed by Schwann cells, and galectin-1 secreted by stellate cells. Additionally, under the influence of TGF-β, CAFs also secrete MMPs, facilitating ECM degradation and the release of bioactive factors [120]. This results in the reconstruction of an ECM that favors tumor growth and proliferation.

### Changes in neural plasticity

Neural plasticity changes induced by pancreatic cancer invasion of peripheral nerves are characterized primarily by glial activation and shifts in the intrapancreatic autonomic to sensory nerve fiber ratio [17]. These neuroplastic changes drive a cascade of histopathological transformations, such as neuropathic hypertrophy and increased neural density. As previously discussed, reciprocal chemotactic interactions between cancer cells and neural components facilitate cancer cell proximity to nerves, enabling adhesion and invasion. Recent studies have revealed that Schwann cells within the pancreas undergo adaptive reprogramming during the early stages of cancer development, fostering protective anti-tumor neuronal responses [121]. Concurrently, injured nerves initiate repair and regenerative mechanisms while secreting a spectrum of molecules that sustain cancer cell proliferation and migratory activity. This feedforward loop—where neural damage promotes tumor aggression, and tumor aggression exacerbates neural injury—epitomizes the dynamic reciprocity within the TME.

Schwann cells ensheath peripheral neurons to form myelin sheaths, a structure critical for rapid nerve impulse conduction. Following peripheral nerve injury, Schwann cells are activated via the c-Jun signaling pathway, transitioning into a reparative state [122]. These non-myelinating repair-state Schwann cells organize into Büngner bands to guide axonal regeneration [97]. In pancreatic cancer, hypoxic conditions and interleukin-6 (IL-6) within the tumor microenvironment are key drivers of Schwann cell activation [123]. During this process, Schwann cells exhibit upregulated activity in the c-Jun, Notch, and MAPK signaling pathways, accompanied by increased expression of GFAP and p75NTR, which collectively drive phenotypic and gene expression reprogramming [64, 122, 124]. Beyond their previously described roles in promoting tumor cell adhesion and migration through upregulation of NCAM1, L1CAM, and TGF-β, Schwann cells have recently been implicated in modulating the immune microenvironment—a mechanism that will be elaborated in later sections.

In addition to Schwann cell activation, another prominent feature of neural remodeling in pancreatic cancer is the altered ratio of intrapancreatic nerve fibers, characterized by a significant reduction in sympathetic innervation. Compared to normal pancreatic innervation, parasympathetic nerve distribution remains largely unchanged, whereas sympathetic innervation is markedly diminished—a phenomenon potentially attributable to cancer cell invasion [125]. Sympathetic nerves have been implicated in promoting tumor progression in multiple cancers (e.g., prostate [126, 127] and breast cancer [128, 129]). In pancreatic cancer, norepinephrine released from sympathetic nerves elevates phosphorylated STAT3 levels in a concentration-dependent manner, activating the β-AR/PKA/STAT3 signaling pathway to enhance cancer cell neural infiltration [75, 130]. However, the paradoxical reduction in sympathetic innervation during pancreatic cancer progression may be linked to an underappreciated immunomodulatory and tumor-restrictive role of sympathetic nerves. Contrary to previously reported pro-tumor effects, recent studies suggest that sympathetic nerve ablation exacerbates tumor growth and metastasis [131]. Selective sympathetic denervation experiments reveal that the loss of sympathetic nerves induces the accumulation of immunosuppressive macrophages, thereby facilitating tumor immune evasion. This apparent duality in sympathetic nerve function leads us to hypothesize three key underlying mechanisms: First, the effects are stage-dependent, with pro-tumorigenic signaling dominating in early-stage disease and immunomodulatory anti-tumor effects prevailing in advanced stages. Second, anatomical heterogeneity exists, whereby sympathetic nerve fibers in peritumoral versus intratumoral regions exhibit distinct and even opposing functions. Third, functional diversity is present among different sympathetic nerve subpopulations. Elucidating the molecular triggers of selective sympathetic degeneration and the context-specific roles of various nerve subtypes remains a critical unmet challenge, which will provide a theoretical foundation for the development of safe and effective neural-targeted therapeutic regimens.

Taken together, these four interconnected processes form a self-reinforcing vicious cycle that drives progressive perineural invasion in PDAC, rather than operating as discrete, sequential events. Reciprocal chemotaxis initiates the physical contact between tumor cells and nerves, generating signals that promote tumor cell adhesion to the nerve sheath and trigger extracellular matrix remodeling. In turn, ECM degradation removes physical barriers to invasion and releases sequestered growth factors that further amplify chemotactic signaling. Finally, tumor-induced neural plasticity reprograms Schwann cells and alters the balance of autonomic innervation, creating a permissive neural niche that sustains and accelerates all three preceding processes. Shared signaling molecules, including TGF-β, MMPs, and NGF, act as critical regulatory hubs that coordinate crosstalk between these stages, explaining why single-target therapies have shown limited clinical efficacy. This integrated mechanistic framework not only provides a comprehensive understanding of PNI pathogenesis but also identifies vulnerable points in the cycle that can be exploited for therapeutic intervention.

## Neuro-immune interaction mechanisms in the tumor microenvironment of PDAC

**Fig. 5:**
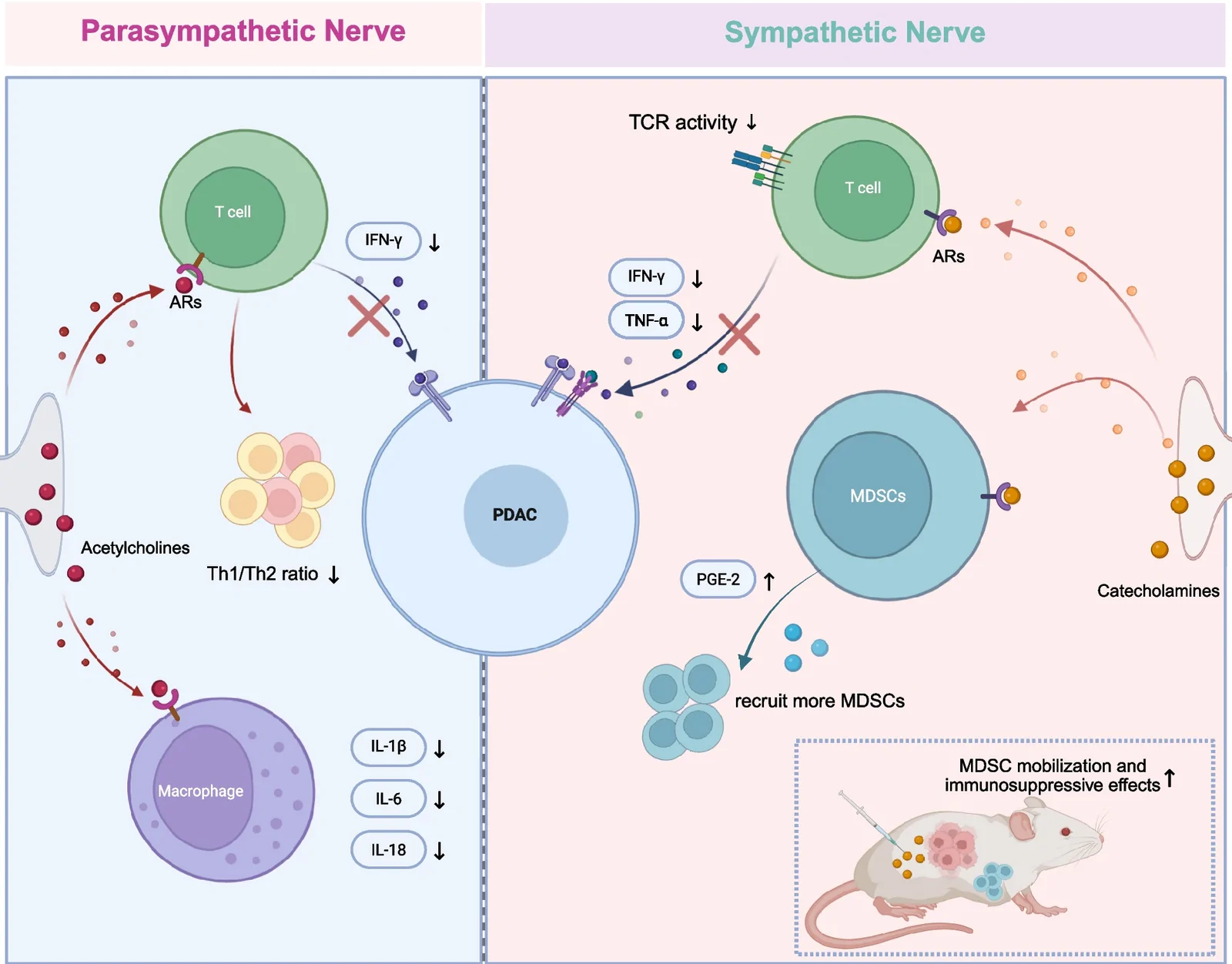
The crosstalk between sympathetic nerve, parasympathetic nerve, and PDAC TME. Sympathetic catecholamines inhibit the anti-tumor function of T cells via ARs, while simultaneously increasing the oxidative phosphorylation of MDSCs and recruiting more MDSCs via PGE2. Notably, in tumor-bearing mouse models, catecholamines exhibit a more pronounced effect on enhancing the mobilization of MDSCs and their immunosuppressive functions. The impact of the parasympathetic nervous system on the PDAC tumor immune microenvironment exhibits a dual role: on one hand, the parasympathetic nervous system impairs the recruitment of CD8^+^ T cells via CCL5 and reduces the Th1/Th2 ratio; on the other hand, ACh inhibits the release of pro-inflammatory cytokines from macrophages. PGE2: Prostaglandin E2

The immune system is dually regulated by both the central nervous system (CNS) and the peripheral nervous system (PNS) [132, 133]. The CNS modulates immune function via hormones secreted by endocrine organs, whereas in the PDAC tumor microenvironment, the PNS exerts localized influence through neuro-immune crosstalk [130]. This regulatory interplay involves contributions from sympathetic nerves, parasympathetic nerves and glial cells (Table 1) [133, 134]. In fact, the changes in the tumor immune microenvironment are extremely complex, and the regulation of the tumor immune microenvironment by nerve cells also has duality. On one hand, nerve cells may promote the inhibitory effect of immune cells on tumors; on the other hand, with the progression of the tumor, the role of nerve cells in promoting tumor immune escape gradually takes the dominant position. The tumor immune microenvironment in PDAC is predominantly populated by immune cells such as T cells, macrophages, myeloid-derived suppressor cells (MDSCs), dendritic cells, and natural killer (NK) cells [135]. A study using next-generation sequencing and immunophenotypic classification has identified three immune subtypes in PDAC, with "immune escape" accounting for 54% and "immune exhaustion" comprising 11% [136]. This "cold" phenotype of the PDAC immune microenvironment is attributed to multiple complex factors, including tumor cell-involved signaling pathways, as well as the extracellular matrix deposition and hypoxic microenvironment we previously discussed, which hinder T cell infiltration. The following sections will explore how nerve cells influence immune cells within the microenvironment (Fig. 5).

**Table 1:** Context-dependent functional heterogeneity of nerve subtypes in PDAC

Neuronal subtype	Functional phenotype	Core molecular mechanism	Immune microenvironment modulation	Experimental evidence	References
Sympathetic adrenergic nerves	Pro-tumorigenic	Activates PKA/STAT3 signaling via β2-AR to enhance tumor cell neural invasiveness; forms a β2-adrenergic-neurotrophin feedforward loop to induce NGF/BDNF secretion and promote innervation; mediates CD8^+^ T cell exhaustion via β1-AR	Promotes MDSC recruitment and immunosuppressive function; impairs CD8^+^ T cell effector activity; drives T cell exhaustion	In vitro cell co-culture assays; KPC genetically engineered mouse models; expression validation in human PDAC tissues	[62, 75, 137–141]
	Tumor-suppressive	Sympathetic axonal sprouting locally inhibits polarization of CD163^+^ M2 macrophages; sympathetic denervation shifts macrophages toward an immunosuppressive phenotype and accelerates tumor growth	Inhibits M2-like polarization of tumor-associated macrophages; maintains local anti-tumor immunity	Selective sympathetic denervation in KIC genetically engineered mouse models	[131]
Parasympathetic cholinergic nerves	Pro-tumorigenic	Acetylcholine downregulates CCL5 via HDAC1 to suppress CD8^+^ T cell recruitment; dose-dependently inhibits IFN-γ secretion by CD8^+^ T cells and reduces the Th1/Th2 ratio	Impairs CD8^+^ T cell infiltration and effector function; facilitates tumor immune evasion	In vitro T cell functional assays; murine PDAC tumor models; expression validation in human PDAC tissues	[119]
	Tumor-suppressive	Inhibits release of pro-inflammatory cytokines (TNF-α, IL-1β) from macrophages via α7nAChR; CHRM1 signaling directly suppresses tumor cell proliferation, stemness and liver metastasis, and reduces CD11b^+^ myeloid cell infiltration	Inhibits pro-inflammatory and pro-tumor activity of tumor-associated macrophages; attenuates chronic inflammation-driven tumor progression	Subdiaphragmatic vagotomy mouse models; genetically engineered mouse models	[142–144]
Sensory peptidergic nerves	Pro-tumorigenic	Tumor-secreted factors induce sensory neuron reprogramming; CGRP^+^ sensory neurons are reduced and switch to pro-tumorigenic factor secretion; the CCL2/CCR4 axis drives tumor cell migration and immune cell recruitment; GCSF/GMCSF stimulate CGRP release to mediate neurogenic inflammation	Recruits immune cells to the perineural microenvironment; participates in perineuritis and formation of the immunosuppressive niche	Trace-n-seq retrograde axonal tracing combined with scRNA-seq; murine models; human PDAC tissue validation	[7, 145–148]

### Sympathetic nerve signaling and β-adrenergic-driven immunosuppression

The proliferation and functional activity of T cells are modulated by sympathetic catecholamines, such as adrenaline and noradrenaline, which predominantly suppress their anti-tumor efficacy [149, 150]. T cells express both α and β adrenergic receptors (ARs) [137]. Activation of the β2-AR signaling pathway in CD8^+^ T cells by adrenaline reduces T cell receptor (TCR) activity, downregulates tumor necrosis factor-α (TNF-α) and interferon-γ (IFN-γ), and consequently impairs their anti-tumor effector functions [151, 152]. Concurrently, β-AR signaling inhibits the motility of both CD8^+^ and CD4^+^ T cells, diminishing their migratory capacity and weakening immune responses. Notably, when CD8^+^ T cells enter an exhausted state, they upregulate β1-AR expression and cluster around sympathetic nerves in a β1-AR-dependent manner [138]. Catecholamines released from these nerves further suppress cytokine production and proliferation in exhausted CD8^+^ T cells. Additionally, catecholamines stimulate angiotensin II type 1 receptors (AT1Rs) on CD4^+^ T cells, inhibiting T-bet-dependent differentiation of CD4^+^ helper T cells into pro-inflammatory Th1 lineages, thereby attenuating cytotoxic anti-tumor responses [153]. In pancreatic cancer models resistant to immune checkpoint blockade (ICB), combined administration of β-AR antagonists and ICB agents significantly enhances CD8^+^ T cell-mediated anti-tumor activity and mitigates T cell exhaustion [154].

The mechanisms by which sympathetic nerves influence TAMs within the PDAC microenvironment remain poorly understood. In other malignancies, sympathetic nerves have been shown to recruit and promote macrophage infiltration, thereby facilitating tumor immune evasion and progression. For instance, in ovarian cancer, adrenergic signaling stimulates tumor cells to produce monocyte chemoattractant protein-1 (MCP-1), which recruits macrophages and enhances their infiltration [150]. In breast cancer, β-adrenergic stimulation induces the infiltration and polarization of pro-tumorigenic macrophages in the TME, driving tumor progression and angiogenesis [155]. Additionally, β-adrenergic activation increases monocyte mobilization and macrophage infiltration into pre-metastatic lungs, promoting pulmonary metastasis [156]. However, in PDAC, sympathetic denervation paradoxically enhances the infiltration of CD163^+^ M2 macrophages, suppresses T cell activity, and accelerates tumor growth [131]. This suggests that sympathetic nerves in PDAC may exert context-dependent anti-tumor effects by modulating macrophage polarization.

MDSCs are a population of suppressive cells originating from the bone marrow, which exhibit an immunosuppressive phenotype [157]. An increasing body of evidence indicates that MDSCs play a pivotal role in establishing an immunosuppressive microenvironment and facilitating tumor immune escape [158–160]. Sympathetic neurons critically regulate the recruitment and immunosuppressive function of MDSCs within the tumor microenvironment primarily through β-ARs [160]. Both monocytic – MDSCs (M-MDSCs) and polymorphonuclear-MDSCs (PMN-MDSCs) express β-ARs [161]. However, the expression level of β2-ARs on PMN-MDSCs is higher than that on M-MDSCs, making PMN-MDSCs more sensitive to sympathetic nerve signals [139]. When treating mouse models with catecholamines, the percentage of MDSCs in untreated mice shows no significant change, while in tumor bearing mice, MDSC mobilization and immunosuppressive effects are more pronounced, which further indicates the role of the sympathetic nervous system in MDSCs in tumor bearing individuals [139]. Research shows that the expression level of β2-AR in MDSCs increases with the increase of tumor burden [162]. This suggests that as the tumor progresses, the immunosuppressive effect of the sympathetic nervous system in attracting MDSCs will further increase. In addition, adrenergic signaling increases the oxidative phosphorylation of MDSCs, thereby increasing the level of prostaglandin E2 (PGE2) through over expression of cyclooxygenase 2 [139]. PGE2 is also a signal for recruiting MDSCs in the microenvironment, which will recruit more MDSCs [163]. In breast cancer, it has been found that in addition to directly recruiting MDSCs through β-ARs, the sympathetic nervous system can also up-regulate the IL-6 and JAK/STAT3 signaling pathways, further promoting the accumulation of MDSCs in the tumor microenvironment [140]. The latest research also indicates that adrenergic signaling can activate MDSCs, trigger the STAT3 signaling pathway, lead to metabolic reprogramming of MDSCs, and improve the survival rate of MDSCs by acquiring an anti-apoptotic phenotype [141]. All of the above demonstrate the immunosuppressive effect of the sympathetic nervous system through MDSCs.

### Parasympathetic nerve and cholinergic anti-inflammatory pathways

Based on existing studies, the parasympathetic nervous system exerts dual roles in the PDAC immune microenvironment—both tumor-promoting and tumor-suppressive. In PDAC, T cell activity is also regulated by parasympathetic innervation. T cells express both muscarinic acetylcholine receptors (mAChRs) and nicotinic acetylcholine receptors (nAChRs) [164]. Following tumor-induced nerve invasion, elevated acetylcholine levels impair the ability of PDAC cells to recruit CD8^+^ T cells via histone deacetylase 1 (HDAC1) mediated suppression of C–C motif chemokine ligand 5 (CCL5) [151]. Concurrently, acetylcholine suppresses IFN-γ production in CD8^+^ T cells and reduces the Th1/Th2 ratio, thereby attenuating anti-tumor immunity and promoting tumor growth. However, another study showed that subdiaphragmatic vagotomy leads to TNFα release by TAMs in pancreatic cancer mouse models, promoting increased tumor cell proliferation and migration [142]. The parasympathetic nervous system acts via the α7 nicotinic acetylcholine receptor (α7nAChR) on macrophages, inhibiting the production and release of pro-inflammatory cytokines such as TNF, IL-1β, IL-6, and IL-18, thus highlighting the potential tumor-restrictive role of parasympathetic innervation [143, 165].

The interaction mechanisms between parasympathetic nerves and MDSCs within the tumor microenvironment remain poorly characterized. In murine breast cancer models, electroacupuncture stimulation of the parasympathetic nervous system has been shown to suppress MDSC accumulation, downregulate pro-inflammatory cytokines, and attenuate their immunosuppressive capacity [166]. Conversely, in colitis, acetylcholine upregulates interleukin-10 (IL-10) expression in M-MDSCs and activates the nAChR/ERK pathway to inhibit inflammatory responses [167]. However, the interplay between parasympathetic nerves and MDSCs in PDAC remains to be fully elucidated, necessitating further mechanistic exploration.

### Schwann cells and glial-mediated immune modulation

Upon invasion and damage by tumor cells, Schwann cells undergo a reprogramming process to promote neural repair and regeneration. During this process, Schwann cells also secrete cytokines that influence immune cells. Activated Schwann cells in PDAC secrete CCL2, which recruits inflammatory monocytes via CCR2 receptor binding, driving their differentiation into TAMs with elevated cathepsin B expression [88]. Concurrently, Schwann cells release interleukin-33 (IL-33), stimulating TAMs to produce basic fibroblast growth factor (bFGF) through ST2 receptor/MyD88-NF-κB pathway activation [168]. Cathepsin B facilitates nerve invasion by degrading the perineurium, while bFGF initiates a feedforward loop by reactivating Schwann cells via PI3K-AKT-Myc signaling [88, 168]. This dual mechanism establishes a self-reinforcing cycle that amplifies perineural invasion, highlighting Schwann cells as pivotal orchestrators of neuro-macrophage crosstalk in PDAC progression.

Schwann cells exert multifaceted immunomodulatory effects on NK cells and DCs within the tumor microenvironment. Mechanistically, Schwann cell-derived interleukin-6 (IL-6) activates the STAT6 signaling pathway in NK cells, downregulating the expression of activating receptors NKp30 and NKG2D, thereby suppressing NK cell cytotoxicity [169, 170]. Concurrent secretion of TGF-β further impairs NK cell activity by inhibiting perforin/granzyme B production. In melanoma-bearing murine models, tumor-activated Schwann cells reprogram DCs toward a tolerogenic phenotype characterized by diminished co-stimulatory molecule expression and impaired antigen-presenting capacity, ultimately reducing T cell priming efficiency [171]. These Schwann cell-driven mechanisms collaboratively establish an immunosuppressive niche that facilitates tumor immune evasion.

### Perineural niche as an immune-privileged sanctuary

The perineural niche represents a specialized, anatomically distinct microenvironment surrounding nerve fibers that has evolved to protect neural tissue from excessive immune activation and damage. In the context of PDAC, this physiological immune privilege is hijacked by cancer cells to create a sanctuary that shields them from immune surveillance and therapeutic attack, while simultaneously promoting tumor progression and metastasis [172]. Emerging evidence indicates that the perineural niche is not merely a passive route for tumor dissemination but an actively constructed immunosuppressive domain that integrates physical barriers, soluble mediators, and cellular crosstalk to establish a profoundly immune-evasive state [173, 174].

Nerves within the perineural niche actively suppress local immune responses through the release of neurotransmitters and neuropeptides. Sympathetic nerve-derived norepinephrine (NE) acts via β-adrenergic receptors expressed on T cells to inhibit TCR signaling, reduce the production of pro-inflammatory cytokines such as interferon-γ (IFN-γ) and tumor necrosis factor-α (TNF-α), and impair CTL cytotoxic function [175, 176]. Similarly, parasympathetic nerve-released ACh binds to nicotinic ACh receptors on CD8^+^ T cells, suppressing their recruitment and effector activity [151]. Other neurotransmitters and neuropeptides present in the perineural niche, including gamma-aminobutyric acid (GABA) and vasoactive intestinal peptide (VIP), further contribute to immune suppression by inhibiting T cell activation and promoting the development of regulatory T cells (Tregs) [175, 177].

The perineural niche is selectively enriched with immunosuppressive cell populations that actively inhibit anti-tumor immunity. Schwann cells, the glial cells of the peripheral nervous system, play a central role in this process by secreting CCL2, which recruits CCR2^+^ monocytes from the circulation [86, 87]. These monocytes differentiate into M2-polarized TAMs that produce high levels of IL-10 and TGF-β, potently suppressing T cell and NK cell function. Additionally, Schwann cell-derived IL-33 further polarizes macrophages toward a pro-tumorigenic phenotype and promotes their secretion of growth factors that support tumor growth and neural remodeling. The perineural niche also accumulates MDSCs and Tregs, which are recruited via chemokine gradients including CXCL12 and CCL22, respectively. These cells act synergistically to suppress effector immune cell function and maintain the immunosuppressive state of the niche [88, 178].

Metabolic reprogramming within the perineural niche creates a nutrient-deprived, hypoxic, and acidic environment that further inhibits anti-tumor immunity. Axons within the niche secrete serine, an essential amino acid that is preferentially utilized by PDAC cells for proliferation and survival, particularly under nutrient stress conditions. This metabolic crosstalk depletes local serine levels, limiting its availability to T cells and impairing their activation and effector function [179]. Additionally, the dense stroma surrounding the perineural niche leads to significant hypoxia, which activates hypoxia-inducible factor (HIF) signaling pathways in both tumor and immune cells. Hypoxia promotes the polarization of TAMs toward an M2 phenotype and enhances the immunosuppressive function of MDSCs, while simultaneously inhibiting CTL activity [180]. The high glycolytic activity of PDAC cells also leads to lactic acid accumulation and extracellular acidification, which further impairs T cell function and promotes immune suppression [181].

The immune-privileged nature of the perineural niche has profound clinical implications for PDAC treatment. It serves as a reservoir for residual tumor cells that survive systemic chemotherapy and immunotherapy, leading to local recurrence and distant metastasis. Cancer cells within the perineural niche are protected from immune surveillance and therapeutic agents by both physical barriers and local immunosuppressive mechanisms, making them particularly difficult to eradicate. Furthermore, the immune-suppressive signals emanating from the perineural niche can diffuse into the broader tumor microenvironment, contributing to the global "cold" immune phenotype characteristic of PDAC. This highlights the critical need to develop therapeutic strategies that specifically target the perineural immune niche, disrupt its immune privilege, and render it susceptible to immunotherapy.

## Targeting the neuro-immune-tumor axis

Although pancreatic cancer survival rates have statistically improved over the past decade, the persistently low 5-year survival rate of approximately 13% underscores unmet clinical needs [3]. Current first-line therapies primarily depend on surgical resection and chemotherapy, yet 80% of surgically treated patients develop recurrence and perineural invasion [182]. The intricate crosstalk between the nervous system, immune system, and pancreatic cancer cells forms a dynamic neuro-immune-tumor axis that drives tumor initiation, progression, perineural invasion, therapeutic resistance, and cancer-related pain. Targeting this axis has emerged as a promising therapeutic strategy to not only inhibit tumor growth but also alleviate neuropathic symptoms and improve patient quality of life. Recent preclinical studies have identified multiple actionable targets within this axis, and several clinical trials are currently underway to evaluate the efficacy of neuro-immune targeted therapies in pancreatic cancer (Table 2). Given the high prevalence of neural infiltration, the critical role of tumor-neural interactions in progression, and limitations of existing treatments, developing therapeutics targeting neuro-immune-tumor axis neuro-tumor interactions represents a promising strategic direction for PDAC management.

**Table 2:** Current clinical trials targeting neuro-tumor interactions in PDAC

Role	Trials no.	Phase	Study type	Intervention	Disease setting	Therapeutic rationale	Trial status	PNI relevance	Key limitations
β-AR blocker	NCT05451043	Phase2	Non-randomized, single group assignment, open label	Durvalumab + gemcitabine + nab-paclitaxel + continuous propranolol	Advanced hepatopancreatobiliary tumors (including PDAC)	Blockade of β-adrenergic signaling inhibits sympathetic nerve-mediated immunosuppression and tumor progression; combined with chemoimmunotherapy to enhance antitumor efficacy	Recruiting	Indirect association. Inhibits sympathetic β-AR signaling to reverse CD8^+^ T cell exhaustion and MDSC accumulation, thereby indirectly remodeling the perineural immunosuppressive niche; not designed as a direct PNI-targeted regimen	Basket trial design with limited PDAC-specific sample size; no dedicated PNI pathological or imaging endpoints; open-label design carries inherent bias
	NCT03838029	Phase2	Randomized, multicenter, placebo-controlled, quadruple-blind	Propranolol + etodolac (perioperative 35-day regimen)	Resectable PDAC undergoing curative surgery (perioperative setting)	Perioperative dual inhibition of β-adrenergic and prostaglandin pathways attenuates surgery-induced stress responses, reduces prometastatic transformation and preserves antitumor immunity to lower postoperative recurrence	Enrolling by invitation	Indirect association. Mitigates perioperative sympathetic stress to reduce neurotrophic factor release and immunosuppressive niche formation, with potential to reduce PNI-associated recurrence; no direct PNI intervention endpoint	Single-nation multicenter design with limited population diversity; PNI is an exploratory rather than primary endpoint; long follow-up required to capture PNI-related long-term outcomes
	NCT06145074	Phase2	Randomized, multicenter, placebo-controlled, triple-blind	Preoperative propranolol	Resectable/borderline resectable PDAC (preoperative window)	Preoperative β-blockade alleviates anxiety-related stress responses and explores modulatory effects on protumorigenic transcriptional and immunological phenotypes in tumor tissue	Recruiting	Indirect association. Exploratorily evaluates effects of β-blockade on PNI-related transcriptional phenotypes; mechanistically relevant but not designed to validate direct anti-PNI efficacy	Small pilot sample size (n = 30) with limited statistical power; short preoperative treatment window cannot assess long-term PNI progression; no independent centralized PNI pathological review
Parasympathomimetic agent	NCT05241249	Phase2	Single group assignment, open label	Gemcitabine + nab-paclitaxel + twice-daily bethanechol	Resectable/locally advanced PDAC (neoadjuvant setting)	Activation of muscarinic cholinergic signaling directly suppresses tumor cell proliferation and stemness, modulates the inflammatory microenvironment, and improves R0 resection rate when combined with chemotherapy	Recruiting	Indirect association. Based on preclinical evidence that cholinergic signaling inhibits tumor invasion and inflammation, with potential to suppress PNI progression; PNI is not set as a primary trial endpoint	Single-center single-arm design without control group; PNI assessment is retrospective post-surgery; small sample size (n = 37) with insufficient statistical power for PNI subgroup analysis
TRKA Inhibitor	NCT02568267	Phase2	Non-randomized, parallel assignment, open label, global basket trial	Entrectinib (pan-TRK/ROS1/ALK inhibitor)	Locally advanced/metastatic solid tumors harboring NTRK fusions (including PDAC)	ATP-competitive inhibition of TRK kinases blocks constitutive oncogenic signaling driven by NTRK gene fusions, suppressing tumor proliferation, invasion and survival	Active, not recruiting	Direct target association. TrkA is the core receptor mediating NGF-driven mutual chemotaxis in PNI; the drug directly blocks this signaling axis. However, the trial enrolls by NTRK fusion status rather than PNI status, and PDAC accounts for a very small proportion	Pan-cancer basket design with extremely low PDAC enrollment (< 5%); enrollment based on gene fusion rather than PNI status, cannot directly validate anti-PNI efficacy; does not cover PNI populations with TrkA overexpression but no fusion
	NCT03556228	Phase1	Sequential assignment, open label, dose escalation	VMD-928 (selective allosteric TrkA inhibitor); combination with pembrolizumab	Advanced solid tumors/lymphoma driven by TrkA overexpression (including PDAC)	Selective allosteric inhibition of TrkA blocks downstream MAPK and other pro-invasive pathways in tumors with TrkA overexpression; combination with anti-PD-1 explores synergistic antitumor immunity	Recruiting	Direct target association. Targets the core NGF/TrkA axis of PNI and theoretically directly blocks bidirectional tumor-nerve chemotaxis. However, the trial does not stratify enrollment by PNI status and lacks dedicated PNI efficacy assessment	Phase 1 dose-escalation trial with safety as primary endpoint and limited efficacy data; PDAC is a small subgroup among pan-cancer indications; no PNI-specific biomarker stratification or dedicated endpoint
GDNF/RET inhibitor	NCT04820179	Phase2	Single group assignment, open label	Cabozantinib (multi-target TKI including RET) + atezolizumab	Metastatic, refractory PDAC	Multi-target kinase inhibition (RET/VEGFR/MET) combined with anti-PD-L1 immunotherapy simultaneously suppresses tumor proliferation, angiogenesis and immunosuppressive microenvironment in refractory PDAC	Active, not recruiting	Indirect association. Cabozantinib inhibits RET kinase, which may block GDNF/RET-mediated tumor-neural chemotaxis and invasion. However, as a multi-target combination regimen, the specific anti-PNI effect of RET inhibition cannot be isolated	Single-center small sample size (n≈30); cabozantinib is a multi-target inhibitor, and the anti-PNI effect cannot be dissociated from effects on other pathways; no PNI stratification or dedicated assessment endpoint

Data source: public clinical trial registration databases ClinicalTrials.gov

Recent study have demonstrated that cancer-induced neural damage is a major factor contributing to the suboptimal efficacy of anti-PD-1 therapy across multiple cancer types [14]. This finding suggests that targeting nerve invasion in cancer may serve as a viable strategy to overcome anti-PD-1 resistance in patients with various malignancies. Currently, therapeutic approaches focusing on neuro-tumor interactions and neurotransmitters have achieved progressive advances in multiple cancer settings. In melanoma patients, the use of non-specific β-blockers has been shown to reduce the recurrence rate and improve overall survival in those with metastatic disease who are concurrently receiving immunotherapy [183]. In a clinical trial involving breast cancer patients, preoperative β-blockade with propranolol effectively reduced intratumoral mesenchymal polarization and decreased the expression of biomarkers associated with metastatic potential [184]. Several clinical trials are actively investigating neuro-immune targeted approaches for pancreatic cancer. For adrenergic signaling modulation, a phase II trial is evaluating the combination of propranolol (a nonselective β-blocker) and etodolac (a COX-2 inhibitor) in patients undergoing surgical resection for pancreatic cancer [185]. For cholinergic signaling enhancement, a phase II trial is assessing the safety and tolerability of bethanechol (a muscarinic agonist) in combination with gemcitabine and nab-paclitaxel for advanced pancreatic cancer patients [144]. In addition, multiple clinical trials are also investigating parasympathomimetic agents, TRKA inhibitors, and GDNF/RET inhibitors. Although recent research advances have provided a completely new understanding of the mechanisms underlying perineural invasion in pancreatic ductal adenocarcinoma, and a small number of clinical studies focusing on nerve-tumor interactions are currently underway, there remains a substantial gap between existing research findings and mature therapeutic strategies that can effectively improve the treatment outcomes of PDAC.

### Potential therapeutic targets

**Fig. 6:**
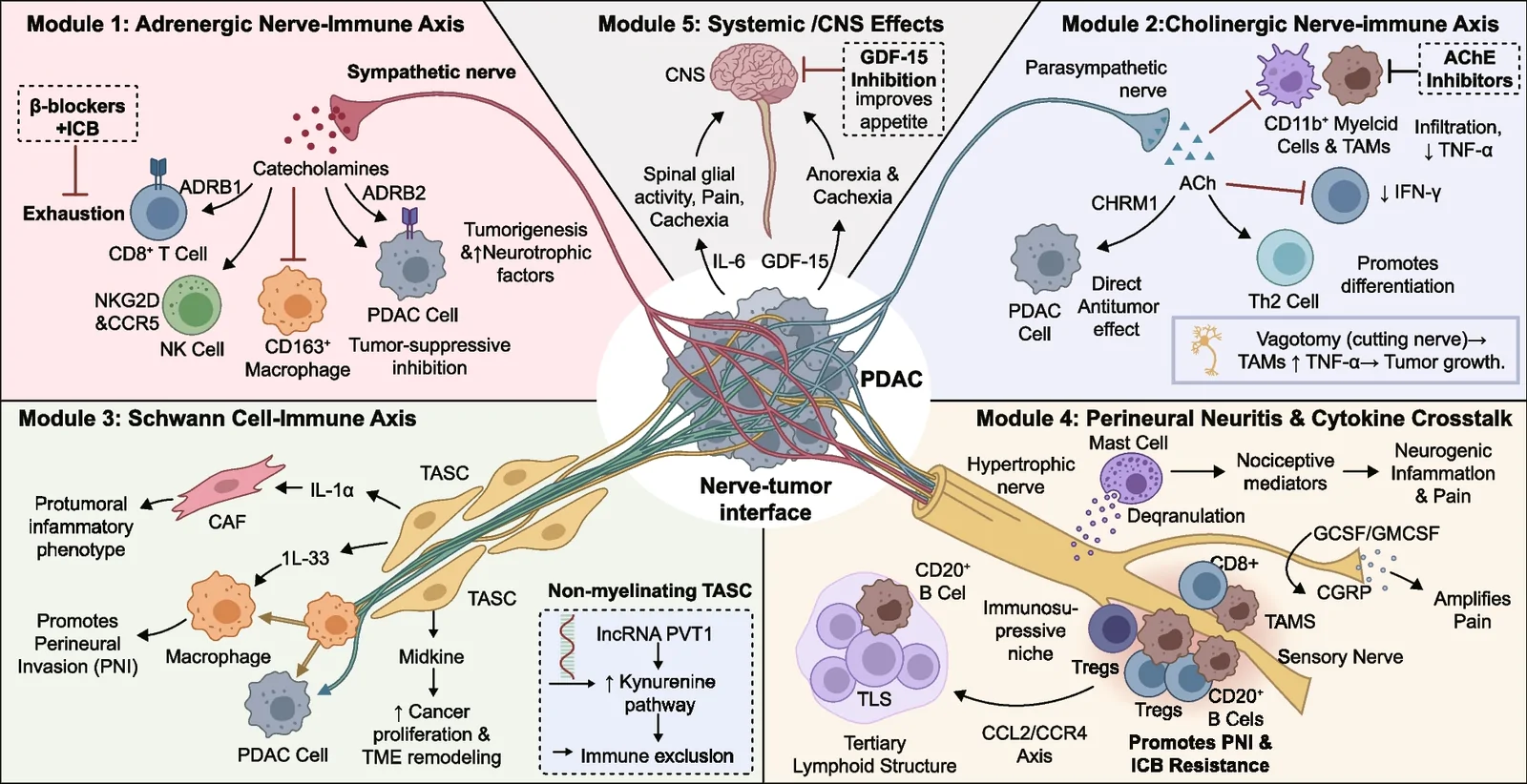
Potential therapeutic targets of the neuro-immune-tumor axis in PDAC. We categorize potential therapeutic targets into five functional modules: Module 1, Adrenergic Nerve-Immune Axis, including β-adrenergic receptors and their regulatory effects on T cells, macrophages and NK cells; Module 2, Cholinergic Nerve-Immune Axis, including muscarinic receptors, acetylcholinesterase inhibitors and vagus nerve-mediated immune modulation; Module 3, Schwann Cell-Immune Axis, including IL-1, IL-33, lncRNA *PVT1* and midkine signaling pathways; Module 4, Perineural Neuritis and Cytokine Crosstalk, including mast cells and multiple immune cell subsets forming the immunosuppressive perineural niche; Module 5, Systemic effects, including neurogenic inflammation, pain and cancer cachexia

The neuro-immune-tumor axis that underpins the formation of the immunosuppressive perineural sanctuary represents a multifaceted therapeutic target pool, with actionable molecules distributed across autonomic nerve signaling, glial cell regulation, perineural inflammation, and cytokine/chemokine crosstalk (Fig. 6). Unlike conventional cytotoxic regimens that primarily target malignant cells, interventions directed at this axis aim to disrupt bidirectional crosstalk among nerves, stromal cells and immune compartments, thereby dismantling the perineural protective niche, attenuating neural invasion, and sensitizing tumors to chemo- and immunotherapy [5]. According to their functional mechanisms and relevance to PNI pathogenesis, candidate targets can be broadly categorized into direct modulators of core PNI pathways and indirect regulators of the perineural immune microenvironment. Below we systematically summarize preclinically validated targets across five functional modules, and evaluate their translational potential and existing limitations (Table 3).

- (1) Adrenergic nerve-immune axis targets
  Β-adrenergic receptors represent key therapeutic targets in this axis. The β1-adrenergic receptor (ADRB1) mediates sympathetic nerve-induced CD8^+^ T cell exhaustion, and combination therapy with β-blockers and immune checkpoint blockade synergistically enhances CD8^+^ T cell antitumor responses [138]. The β2-adrenergic receptor (ADRB2) drives pancreatic tumorigenesis through both direct effects on cancer cells and indirect modulation of neurotrophic factor secretion and immune microenvironment remodeling [62]. Notably, sympathetic innervation also exerts tumor-suppressive effects by locally inhibiting CD163^+^ macrophage subsets, highlighting the context-dependent roles of adrenergic signaling [131]. Additionally, sympathetic nerves regulate NK cell antitumor activity through modulation of NKG2D and CCR5 expression [186].
- (2) Cholinergic nerve-immune axis targets
  The cholinergic nerve-immune axis offers another promising therapeutic avenue. The M1 muscarinic receptor (CHRM1) mediates the direct antitumor effects of acetylcholine, while also reducing CD11b^+^ myeloid cell infiltration and TNF-α production [144]. Acetylcholine further modulates adaptive immunity by dose-dependently inhibiting IFN-γ production by CD8^+^ T cells and promoting Th2 cell differentiation [151]. Indirect activation of cholinergic signaling via acetylcholinesterase inhibitors reduces tumor-associated macrophage infiltration and systemic proinflammatory cytokine levels, whereas subdiaphragmatic vagotomy exacerbates tumor growth through increased TNF-α secretion by tumor-associated macrophages [142, 187].
- (3) Schwann cell-immune axis targets
  Schwann cells, the major glial cells of the peripheral nervous system, are central regulators of the perineural immune microenvironment. Tumor-associated Schwann cells secrete IL-1, which induces cancer-associated fibroblasts to adopt a protumoral inflammatory phenotype [188]. They also release IL-33 to recruit macrophages into the perineural niche, facilitating perineural invasion [168]. Furthermore, tumor-associated non-myelinating Schwann cells express the long noncoding RNA *PVT1*, which promotes the kynurenine pathway and mediates tumor immune exclusion [189]. Midkine signaling from Schwann cells also contributes to both cancer cell proliferation and immune microenvironment remodeling [188].
- (4) Perineural neuritis-related immune targets
  Perineural neuritis, a hallmark of pancreatic neuropathy, is characterized by immune cell infiltration around hypertrophic nerves and is closely associated with abdominal pain [7]. Key immune targets in this context include mast cells, which are specifically enriched in perineural regions and mediate neurogenic inflammation and pain through degranulation and release of nociceptive mediators [145, 193]. Other immune cell subsets involved in perineural inflammation include CD8^+^ cytotoxic T cells, tumor-associated macrophages, and CD20^+^ B cells in tertiary lymphoid structures, which together form an immunosuppressive perineural niche that promotes tumor progression and immunotherapy resistance [146, 190].
- (5) Cytokine/chemokine neuro-immune interaction targets
  Multiple cytokines and chemokines mediate neuro-immune crosstalk in pancreatic cancer. The CCL2/CCR4 axis drives cancer cell migration toward neurons and recruits immune cells to the perineural microenvironment [148]. Granulocyte colony-stimulating factor (GCSF) and granulocyte–macrophage colony-stimulating factor (GMCSF) enhance calcitonin gene-related peptide (CGRP) release from sensory nerves, amplifying neurogenic inflammation and pain [7]. IL-6 activates Schwann cells to modulate spinal glial activity and pain perception, while also mediating cancer cachexia through central nervous system effects [191, 192]. Growth differentiation factor 15 (GDF-15) represents another promising target, as its inhibition improves appetite and physical activity in pancreatic cancer patients through central nervous system modulation [194].

**Table 3:** Potential targets of the pancreatic cancer–neuron–immune interaction and preclinical studies

Target		Preclinical experimental content	Level of evidence	References
Adrenergic Nerve-Immune Axis Targets	ADRB1	Sympathetic nerves mediate CD8^+^ T cell exhaustion in pancreatic cancer via an ADRB1-dependent mechanism; combination therapy with β-blockers and immune checkpoint blockade synergistically enhances CD8^+^ T cell antitumor responses	Supported by in vivo animal models	[138]
	ADRB2	Sympathetic nerves release catecholamines that promote pancreatic epithelial cell tumorigenesis through ADRB2, while inducing NGF/BDNF secretion to enhance innervation and indirectly remodel the tumor immune microenvironment	Validated in human PDAC tissues	[62]
	CD163^+^ macrophages	Sympathetic denervation in KIC mouse models leads to increased numbers of CD163^+^ macrophages, which in turn promote tumor growth and metastasis; sympathetic axonal sprouting exerts antitumor effects by locally inhibiting CD163^+^ macrophage subsets	Supported by in vivo animal models	[131]
	NK cells	Positive stress induced by enriched housing conditions enhances NK cell antitumor immunity via sympathetic nerve-dependent regulation of NKG2D and CCR5 expression	Supported by in vivo animal models	[186]
Cholinergic Nerve-Immune Axis Targets	CHRM1	Cholinergic signaling via CHRM1 not only directly inhibits pancreatic cancer cell proliferation but also reduces CD11b^+^ myeloid cell infiltration, decreases TNF-α levels, suppresses cancer stemness and liver metastasis	Validated in human PDAC tissues	[144]
	Acetylcholine	1. Acetylcholine dose-dependently inhibits IFN-γ production by CD8^+^ T cells and promotes Th2 rather than Th1 cell differentiation, thereby suppressing intratumoral T cell responses. 2. Indirect activation of cholinergic signaling (via acetylcholinesterase inhibitors) reduces tumor-associated macrophage infiltration and serum proinflammatory cytokine levels	Validated in human PDAC tissues	[151, 187]
	TNF-α	Subdiaphragmatic vagotomy promotes pancreatic cancer growth and reduces survival in mice by upregulating TNF-α secretion from tumor-associated macrophages	Supported by in vivo animal models	[142]
Schwann Cell-Immune Axis Targets	IL-1α	Tumor-associated Schwann cells secrete IL-1α, which induces cancer-associated fibroblasts to adopt a protumoral inflammatory phenotype and remodel the immunosuppressive microenvironment	Validated in human PDAC tissues	[188]
	IL-33	Schwann cells release IL-33 to recruit macrophages into the perineural microenvironment, forming a pro-invasive immune niche that supports pancreatic cancer perineural invasion	Validated in human PDAC tissues	[168]
	lncRNA PVT1	Tumor-associated non-myelinating Schwann cells express lncRNA PVT1, which promotes the kynurenine pathway in pancreatic cancer and mediates tumor immune exclusion	Validated in human PDAC tissues	[189]
	Midkine	Schwann cells not only directly promote pancreatic cancer cell proliferation and migration via midkine signaling but also indirectly regulate the recruitment and activation of immune cells in the tumor microenvironment	Validated in human PDAC tissues	[188]
Perineural Neuritis-Related Immune Targets	Mast cells	Mast cells are one of the major infiltrating immune cell subsets in pancreatic perineural neuritis, participating in nerve injury and inflammatory responses	Validated in human PDAC tissues	[145]
	Tumor-associated macrophages	1. Tumor-associated macrophages in pancreatic nerve bundles upregulate CCL7, CXCL12 and CXCL13 chemokines, promoting nerve injury repair and tumor perineural invasion. 2. M2 macrophages in the perineural microenvironment possess immunosuppressive functions and promote tumor immune escape	Validated in human PDAC tissues	[146]
	CD20^+^ B cells	Abundant nerve fibers are present in tertiary lymphoid structures of pancreatic cancer tissues, primarily colocalized with CD20^+^ B cells and participating in local immune regulation	Validated in human PDAC tissues	[190]
Cytokine/Chemokine Neuro-Immune Interaction Targets	CCL2/CCR4 axis	Cancer cell-derived TGF-α induces CCL2 secretion from sensory neurons, which activates migration signals through CCR4 on cancer cells and simultaneously recruits immune cells to the perineural microenvironment	Validated in human PDAC tissues	[148]
	GCSF/GMCSF	GCSF and GMCSF enhance CGRP release from nerves via their receptors, promoting neurogenic inflammation and pain, while also regulating myeloid immune cell differentiation	Supported by in vivo animal models	[7]
	IL-6	IL-6 secreted by pancreatic cancer cells activates Schwann cells to modulate spinal glial activity; IL-6 also mediates cancer cachexia through effects on neurons in the area postrema	Validated in human PDAC tissues	[191, 192]

### Overcoming challenges and emerging strategies

Despite promising preclinical results, translating neuro-immune-tumor axis targeting into clinical practice faces several significant challenges. Surgical or systemic pharmacological denervation disrupts the essential physiological functions of the nervous system, including pancreatic exocrine and endocrine secretion, gastrointestinal motility, and pain sensation. For example, celiac plexus neurolysis, while effective for pain relief, is associated with significant side effects such as diarrhea, hypotension, and neurological deficits, limiting the use of broad neural ablation as a first-line therapy and highlighting the need for tumor-specific neural targeting strategies [195–197].

Current mouse models of pancreatic cancer fail to accurately recapitulate the complex neuroanatomy of human disease. Unlike humans, mice lack large nerve trunks in the pancreas, resulting in the absence of true endoneurial invasion in most autochthonous models [17]. Additionally, the innervation patterns and immune cell composition of mouse pancreatic tumors differ significantly from those of human patients, leading to inconsistent results between preclinical and clinical studies. These limitations have hampered the development and validation of neural-targeted therapies, even though orthotopic allograft and xenograft models can partially mimic tumor spread to the celiac ganglia and spinal cord [198, 199].

The roles of different nerve subtypes in pancreatic cancer are context-dependent and stage-specific. While sympathetic nerves generally promote tumor progression in early-stage disease via β2-adrenergic-neurotrophin feedforward loops, a study in the KIC mouse model showed that sympathetic innervation actually suppresses tumor growth in later stages by inhibiting CD163^+^ macrophage accumulation [62, 131]. Furthermore, higher nerve density has been associated with improved clinical outcomes in some human studies, likely due to the presence of antitumor immune cells in nerve-associated tertiary lymphoid structures [200]. This heterogeneity means that a "one-size-fits-all" approach to neural targeting is unlikely to be effective.

The dense desmoplastic stroma of pancreatic cancer creates a significant physical barrier to drug delivery, particularly to the perineural niche which is often located deep within the tumor [201]. Systemically administered drugs may not reach sufficient concentrations in the perineural region to effectively block cancer-nerve interactions, leading to treatment failure. Additionally, chemotherapy itself induces neural remodeling in the pancreatic tumor microenvironment: 5-fluorouracil triggers endoplasmic reticulum stress in cancer cells, leading to proBDNF production and increased neurite outgrowth, and also activates a neural-like progenitor program in pancreatic cancer cells, enhancing their invasive potential [202, 203].

To address these challenges, several innovative therapeutic strategies are being developed to target the neuro-immune-tumor axis with greater precision and efficacy. Localized delivery of neurotoxic agents directly into the tumor or peritumoral region allows for selective ablation of tumor-associated nerves while sparing normal innervation. Preclinical studies have shown that intratumoral injection of botulinum toxin or targeted neurotoxic nanoparticles effectively reduces tumor innervation and growth without causing systemic side effects, making this approach particularly promising for locally advanced pancreatic cancer where surgical resection is not feasible [204]. Novel techniques such as 3D panoramic histology with tissue clearing, viral tract tracing, and spatial transcriptomics are revolutionizing our ability to map pancreatic innervation at single-cell resolution [205, 206]. These tools allow researchers to identify specific nerve subtypes and molecular pathways that drive tumor progression, enabling the development of more precise neural-targeted therapies. In the future, these imaging techniques may also be used to guide surgical denervation, ensuring complete removal of tumor-infiltrated nerves while preserving normal physiological function.

Recent studies have revealed that intratumoral axons provide serine to pancreatic cancer cells, supporting their growth and survival in the nutrient-deficient tumor microenvironment [66]. Serine deprivation further enhances tumor innervation by increasing NGF secretion, creating a vicious cycle of metabolic dependency and nerve invasion. Targeting this neuro-metabolic axis—either by inhibiting serine uptake by cancer cells or blocking axonal serine release—represents a novel therapeutic approach to starve pancreatic cancer cells.

Pancreatic cancer exerts profound effects on the central nervous system, leading to cachexia, anorexia, cognitive decline, and altered pain perception [207, 208]. Targeting these central effects can improve patient quality of life and may also enhance antitumor immunity. In clinical settings, targeting GDF-15, a cytokine linked to cancer cachexia, leads to improved appetite and increased physical activity in pancreatic, lung, and colorectal cancer patients [194]. In preclinical models, Lipocalin 2 was shown to mediate both appetite suppression and cognitive decline under cancer cachectic conditions, making it a potential target for reversing these symptoms [209, 210].

In conclusion, targeting the neuro-immune-tumor axis represents a paradigm shift in pancreatic cancer treatment. While significant challenges remain, advances in our understanding of cancer neuroscience and the development of novel targeted therapies hold great promise for improving outcomes for this devastating disease. Future success will require the development of more accurate preclinical models that recapitulate human nerve invasion, the identification of predictive biomarkers for patient stratification, and the design of rational combination therapies that simultaneously target neural, immune, and metabolic pathways.

## Conclusion and future perspectives

### Multi-omics insights into neuro-tumor interactions in PDAC

Multi-omics technologies have been widely implemented in PDAC research. Recent investigations increasingly prioritize integrative multi-omics analyses over single-omics approaches, as the latter only partially elucidate the complex interaction networks within the tumor ecosystem, while integrative analyses provide multidimensional insights into intratumoral mechanisms. For instance, single-cell RNA sequencing (scRNA-seq) resolves tumor heterogeneity at cellular resolution but lacks spatial context [211, 212]. This limitation is addressed by coupling scRNA-seq with spatial transcriptomics [212, 213]. Ashiq Masood et al. employed spatially resolved transcriptomics and ecotype analysis to map the PDAC immune microenvironment, revealing significant ecotype enrichment disparities across anatomical regions [214]. Notably, invasive border ecotypes harbored both tumor-promoting and tumor-suppressive cellular populations, suggesting these niches as potential immunotherapeutic targets. Chandwani et al. integrated CosMx Spatial Molecular Imaging with genomic profiling to delineate KRAS mutation subtype-specific variations at cellular, molecular, and microenvironmental levels, offering novel strategies for patient stratification and personalized therapy [215]. Additionally, Tian et al. developed TMEPro, a clinical-grade multidimensional proteomic strategy, which uncovered a universal shedding mechanism of plasma membrane proteins in PDAC tumors [216]. Their work demonstrated that matrix metalloproteinase-mediated shedding of the AXL receptor tyrosine kinase ectodomain introduces an additional regulatory layer for intercellular signaling within the PDAC TME.

Recent advances in multi-omics technologies have provided novel insights into the mechanisms of neural infiltration in PDAC. High-throughput sequencing and protein microarray analyses revealed that PDPN + CAFs promote nerve invasion by delivering PIAT to tumor cells via exosomes, which inhibits YBX1 ubiquitination and enhances its binding to nerve invasion-related mRNAs through m5C methylation, thereby stabilizing these transcripts [49]. Further studies identified three distinct PDAC biotypes: glandular-like and transitional subtypes exhibit gene expression patterns associated with endodermal differentiation and ECM synthesis, respectively, while the undifferentiated subtype shows upregulated expression of stem/progenitor cell regulators and neuroactivity-related proteins [48]. Notably, tumor-derived secreted frizzled-related protein 2 (sFRP2) enhances ECM deposition, tumor cell migration, and neural infiltration. Additionally, the newly developed Trace-n-seq technology, integrating retrograde axonal tracing and single-cell transcriptomics, uncovered PDAC-driven neuronal reprogramming [147]. Tumor-secreted Slit2 and Lin28b induced a 2.3-fold increase in tyrosine hydroxylase expression in sympathetic neurons to stimulate angiogenesis, while CGRP^+^ sensory neurons were reduced by 40%, switching to pro-tumorigenic factor secretion. Collectively, these multi-omics findings systematically delineate the multilayered regulatory networks underlying nerve invasion, and provide an unprecedented molecular resolution for decoding the cellular architecture and signaling crosstalk of the immunosuppressive perineural sanctuary.

### Artificial intelligence and multi-omics integration: advancing PNI diagnosis and mechanistic discovery

Currently, AI and deep learning applications in PDAC PNI research primarily focus on diagnostic assistance [217]. Pathological evaluation of PNI requires meticulous examination, as even minute neural infiltration can shift PNI status from negative to positive [8, 218]. AI-assisted diagnostic tools show promise in improving accuracy and efficiency. In one study, a histopathological computational analysis algorithm was trained on 260 manually annotated nerve-tumor images to develop a PDAC PNI detection model, achieving specificities of 78% for nerve detection and 85% for tumor identification [219]. Although limited by small training cohorts and room for specificity improvement, this represents a pioneering effort. Another study involving 1,065 patients developed a fully automated CT-based AI model for extrapancreatic nerve invasion detection and risk stratification [220]. The model demonstrated robust performance in validation cohorts, with mean Dice similarity coefficients of 0.86 for tumor segmentation and 0.81 for vascular mapping. These AI-driven innovations are poised to enhance clinical detection and characterization of PNI in PDAC.

The convergence of AI and multi-omics technologies represents the most promising frontier in PDAC PNI research. Modern high-throughput technologies have generated vast multidimensional datasets spanning genomics, transcriptomics, proteomics, and spatial biology, but decoding the embedded biological insights into neuro-tumor-immune crosstalk remains a formidable challenge [221, 222]. Advanced AI algorithms, particularly foundation models, are uniquely suited to integrate these heterogeneous datasets and unravel the complex regulatory networks underlying PNI.

#### Core research strategies

##### Multimodal reconstruction of neuro-tumor-immune interfaces

Multimodal reconstruction of neuro-tumor-immune interfaces represents the primary strategic application of AI foundation models in PNI research. Integration of spatial transcriptomics, high-resolution proteomics, and digital pathology imaging enables computational reconstruction of the dynamic cellular and molecular architecture at the tumor-nerve interface. This approach resolves the spatial organization of Schwann cells, neurons, immune cells, and cancer cells, elucidating how their reciprocal interactions establish and maintain the immunosuppressive perineural niche [223].

##### Cross-omics identification of dual-function PNI-immune targets

Deep learning algorithms analyze integrated multi-omics datasets to identify key molecular regulators that concurrently drive both nerve invasion and immune evasion. These include extracellular matrix remodeling enzymes, axon guidance molecules, and secreted factors that modulate MDSC recruitment and TAM polarization, representing high-priority therapeutic targets that simultaneously disrupt two critical hallmarks of PDAC progression [224].

##### AI-optimized rational combination therapy design

AI-driven therapeutic pipelines simulate the effects of neural-targeted agents in combination with immunotherapies, identifying synergistic regimens that disrupt protumoral neuro-immune crosstalk while preserving endogenous antitumor immunity. For example, models can predict optimal pairing of neurokinin-1 receptor antagonists with colony-stimulating factor 1 receptor inhibitors to deplete perineural macrophages and restore T cell function [225]. AI-optimized rational combination therapy design provides a computational framework for translational regimen development.

#### Critical challenges

##### Scarcity of standardized multimodal PNI datasets

Scarcity of standardized multimodal PNI datasets represents the foremost bottleneck limiting the clinical translation of AI-multi-omics approaches. There is a critical lack of large, multi-center datasets that integrate matched histopathological, radiological, and multi-omics data from well-annotated PNI-positive PDAC specimens. Most existing datasets are small, single-center, and lack standardized acquisition protocols, limiting the generalizability of trained AI models.

##### Biological interpretability gap of black-box models

Biological interpretability gap constitutes a second fundamental challenge. Current AI models often function as "black boxes," generating predictions that cannot be easily linked to underlying biological mechanisms. This is particularly problematic in PNI research, where understanding the causal relationships between molecular alterations and phenotypic outcomes is essential for therapeutic development.

##### Context-dependent neuronal subtype heterogeneity

Context-dependent neuronal subtype heterogeneity adds a further layer of complexity to AI modeling. The paradoxical and stage-specific roles of different neuronal subtypes (sympathetic, parasympathetic, and sensory) in PDAC progression create significant modeling challenges. AI models must account for this cellular heterogeneity and context-dependent function to avoid generating misleading or biologically irrelevant predictions.

#### Proposed solutions

Establishment of multi-center neurocentric data consortia is proposed as the foundational solution to address data scarcity. Collaborative networks should be formed to aggregate and standardize multimodal data from PNI-positive PDAC specimens, with uniform protocols for sample collection, processing, and annotation. This will create the large, high-quality datasets required to train robust and generalizable AI models.

Development of biologically constrained interpretable AI architectures is critical to bridge the mechanistic gap. Future AI models should incorporate prior biological knowledge—such as known signaling pathways and cellular interaction networks—into their design, enabling them to generate mechanistically interpretable predictions rather than purely correlative outputs. This will bridge the gap between computational predictions and experimental validation.

Implementation of a standardized prediction-validation workflow is essential to ensure clinical translatability of AI discoveries. A rigorous, multi-stage validation pipeline should be established where AI-identified candidate targets and biomarkers are systematically tested using in vitro co-culture systems, in vivo mouse models, and independent clinical cohorts. This will ensure that AI discoveries are biologically relevant and clinically actionable, accelerating the translation of basic research findings into patient benefit.

### Future research directions and translational priorities

**Fig. 7:**
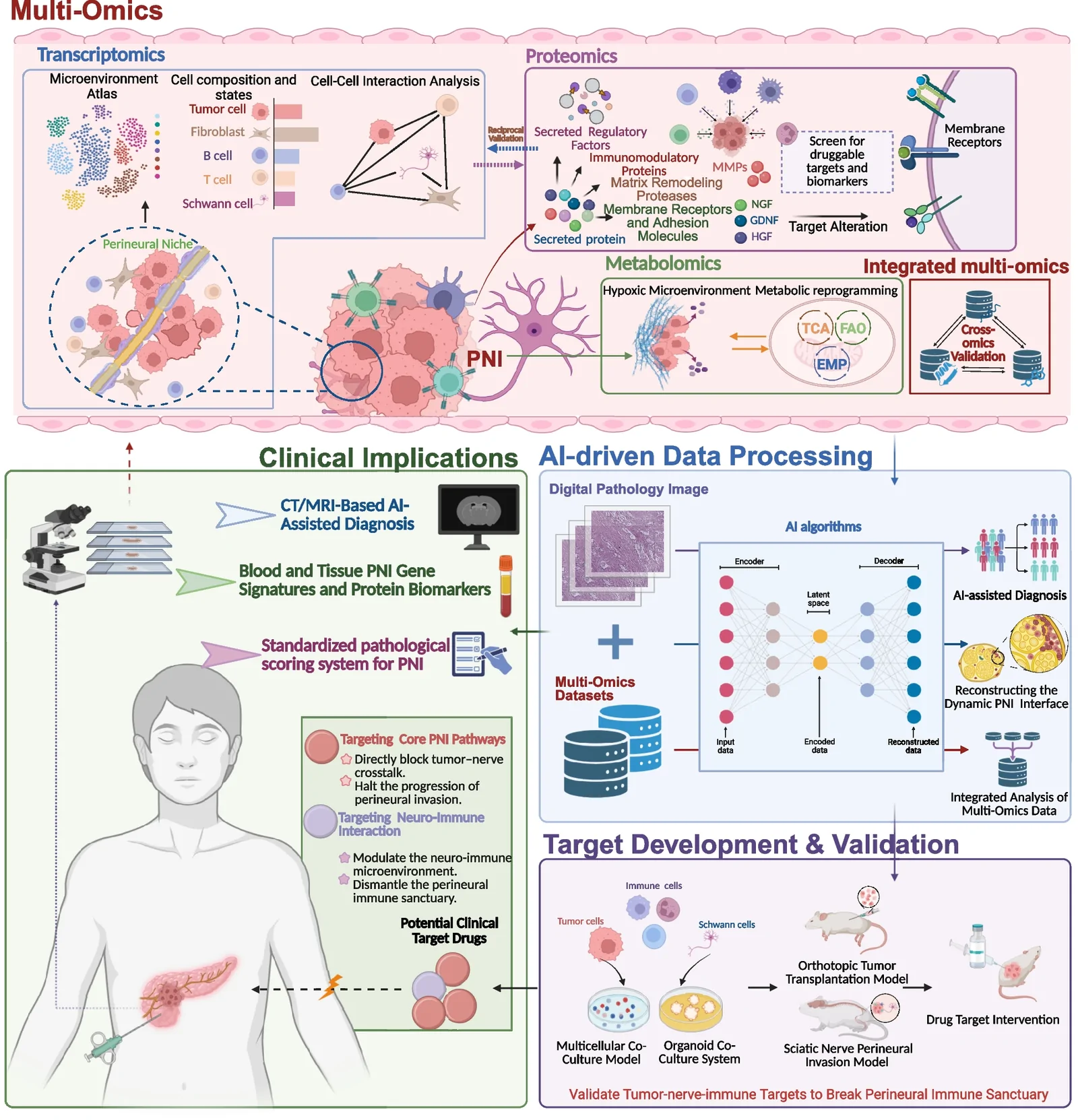
Translational roadmap for dismantling the immunosuppressive perineural sanctuary in Pancreatic Ductal Adenocarcinoma: From multi-omics profiling to AI-empowered precision neuroimmune therapy. This figure depicts a four-stage, closed-loop translational pipeline for decoding PNI in PDAC and developing precision interventions targeting the neuro-immune-tumor axis, centered on the core conceptual framework of the immunosuppressive perineural sanctuary. The first stage is multi-omics molecular profiling of clinical PNI specimens. Transcriptomic analysis (single-cell and spatial transcriptomics) constructs a comprehensive cellular atlas of the perineural microenvironment, quantifies the abundance of diverse cell subtypes, and delineates intercellular crosstalk networks across the tumor-nerve interface. Proteomic profiling characterizes four classes of functional proteins in PNI lesions: Membrane receptors and adhesion molecules, secreted chemotactic and regulatory factors, matrix-remodeling proteases, and immune regulatory proteins. Integrated with transcriptomic data, proteomic analysis provides orthogonal validation for transcript-level findings, maps ligand-receptor interaction networks among neural, tumor and immune compartments, identifies functional driver molecules, and prioritizes druggable targets and clinical stratification biomarkers. Metabolomics further deciphers the specialized metabolic microenvironment of the perineural niche, including nutrient deprivation, acidic metabolite accumulation and immunosuppressive metabolic mediators. Collectively, the three omics layers are integrated to generate a unified molecular signature of the perineural immune sanctuary. The second stage is AI-driven data processing and target mining. Coupled with digital histopathological images, AI-based computational pipelines perform three core tasks: AI-assisted pathological diagnosis and quantitative scoring of PNI, dynamic reconstruction of the tumor-nerve-immune interface at spatial resolution, and integrative multi-omics data analysis to prioritize candidate interventional targets and predict synergistic combination regimens. The third stage is preclinical functional validation. Candidate targets and therapeutic regimens are systematically screened and validated via in-vitro multicellular co-culture assays and in-vivo animal models, to verify their efficacy in suppressing perineural invasion, remodeling the immune microenvironment and inhibiting tumor progression. The fourth stage is clinical translation and precision application. The integration of multi-omics and artificial intelligence enables three clinical implementations: CT/MRI-based AI-assisted diagnosis of extrapancreatic nerve invasion, blood and tissue-based PNI gene signatures and protein biomarkers for patient stratification, and standardized pathological scoring system for PNI. Potential clinical targeted agents are explicitly categorized into two tiers: Agents targeting core PNI pathways to directly block tumor-neural crosstalk, and agents targeting neuro-immune interactions to dismantle the immunosuppressive perineural sanctuary and reverse immunotherapy resistance. Clinical findings and real-world evidence are iteratively fed back to basic multi-omics research to form a closed-loop optimization system

Building on the unified four-stage mechanistic model of PNI and the unifying conceptual framework of the immunosuppressive perineural sanctuary in PDAC, we suggest five prioritized research directions for the next 3–5 years and put forward a field-oriented roadmap integrating advances in multi-omics and artificial intelligence (Fig. 7), with the overarching goal of translating mechanistic insights of PNI into tangible clinical benefit for PDAC patients.

At the foundational level, the establishment of a standardized pathological scoring system for PNI remains the most pressing priority for the field. Current clinical assessment of PNI lacks uniform quantitative criteria, which hinders consistent patient stratification and cross-study comparability. A consensus scoring system should incorporate quantitative parameters including nerve invasion depth, circumferential nerve involvement ratio, perineural immune cell infiltration density, and intrapancreatic nerve remodeling degree, to enable reproducible prognostic stratification of PNI severity.

Building on standardized diagnostic criteria, large-scale PNI-high spatial transcriptomic cohorts are essential to map the human perineural immune niche at single-cell resolution. Multi-center collaborative efforts should prioritize spatial multi-omics profiling of well-annotated PNI-positive PDAC clinical specimens, to delineate the cellular composition, molecular signatures and spatial organization of the perineural sanctuary in human disease, and to identify clinically relevant biomarkers and therapeutic targets validated in patient tissues rather than only in preclinical models.

With expanded clinical cohorts and multi-omics resources, functional validation of neuroimmune targets linked to immunotherapy resistance emerges as a high translational priority. While multiple neuro-immune axes have been implicated in PNI, their causal roles in mediating anti-PD-1 resistance remain insufficiently validated. Functional studies using genetically engineered mouse models and human tissue validation should systematically evaluate how perineural niche components drive immunotherapy failure, and prioritize targets whose blockade can sensitize PNI-high tumors to immune checkpoint inhibition.

To enable reliable and physiologically relevant target validation, advanced organoid-neuron-immune co-culture systems are needed to complement conventional preclinical platforms. Traditional two-dimensional co-cultures and conventional mouse models fail to fully recapitulate the three-dimensional architecture and multicellular complexity of the human perineural sanctuary. Advanced co-culture models integrating PDAC organoids, primary neurons, Schwann cells and immune cell subsets will enable more reliable target validation and drug screening, bridging the gap between basic discovery and clinical testing.

Ultimately, the translation of these mechanistic and preclinical findings relies on PNI-stratified prospective clinical trial design to generate high-level clinical evidence. Future clinical trials evaluating neuroimmune combination therapies should prospectively stratify patients by PNI status and perineural immune signatures, to test whether PNI-high subgroups derive greater benefit from neuro-immune targeted regimens. Such trial designs will enable precision patient selection and generate high-level evidence to support PNI-guided precision therapy.

In conclusion, the intricate crosstalk among the nervous system, immune system and pancreatic cancer cells represents a fundamental and long underappreciated dimension of PDAC biology. This review synthesizes current mechanistic evidence and proposes the unified four-stage mechanistic model of PNI and the immunosuppressive perineural sanctuary as a unifying conceptual framework to understand how PNI drives tumor recurrence, cancer-associated pain, systemic immune evasion and therapeutic resistance. By decoding the multilayered mechanisms of neuro-tumor-immune interactions and leveraging integrative multi-omics and AI technologies, the field is poised to develop novel therapeutic strategies that dismantle the perineural sanctuary, reverse immune suppression, and ultimately improve outcomes for patients with this devastating disease. The roadmap outlined here provides a structured path forward, emphasizing that the future of PDAC treatment lies in a holistic approach targeting not only malignant cells but the entire neuro-immune-stromal ecosystem that supports tumor progression.

## Data Availability

Not applicable.
